# From Fundamentals of Laser-Induced Breakdown Spectroscopy to Recent Advancements in Cancer Detection and Calcified Tissues Analysis: An Overview (2015–2025)

**DOI:** 10.3390/molecules30214176

**Published:** 2025-10-24

**Authors:** Muhammad Mustafa Dastageer, Khurram Siraj, Johannes David Pedarnig, Dacheng Zhang, Muhammad Qasim, Muhammad Shahzad Abdul Rahim, Saba Mushtaq, Qaneeta Younas, Bareera Hussain

**Affiliations:** 1 Laser & Optronics Centre, Department of Physics, University of Engineering and Technology (UET), Main Campus, G.T. Road, Lahore 54890, Pakistan; 2022phdphy8@student.uet.edu.pk (M.M.D.); 2021phdphy3@student.uet.edu.pk (M.Q.); 2017phdphy04@student.uet.edu.pk (M.S.A.R.); 2019phdphy02@student.uet.edu.pk (S.M.); 2020phdphy3@student.uet.edu.pk (Q.Y.); 2022phdphy10@student.uet.edu.pk (B.H.); 2 Institute of Applied Physics, Johannes Kepler University Linz, A-4040 Linz, Austria; 3 School of Optoelectronic Engineering, Xidian University, 2 South Taibai Road, Xi’an 710071, China; dch.zhang@xidian.edu.cn

**Keywords:** LIBS, cancer diagnosis, calcified tissues, matrix effects, analytical chemistry, AI models, hybrid techniques, plasma parameters

## Abstract

Laser-induced breakdown spectroscopy (LIBS) is a promising elemental analysis technique that has rapidly evolved in numerous fields, including biomedical research and medical sciences, over the last two decades. In combination with other methods, it has the potential to examine complex biological structures and their species distributions. The present work first develops the basic understanding of LIBS and then reviews its evolution in oncological diagnosis and calcified tissue analysis from medical perspectives over the last 11 years. LIBS can potentially improve early cancer detection and monitor treatment outcomes, ultimately enhancing patient care and diagnosis. It has effectively differentiated between malignant and normal tissues and also classifies cancer stages and types based on disease severity. Its applications for categorising and identifying calcified tissues are attractive for inspecting minerals, while soft tissue is more challenging, given the potential for significant matrix effects. This review article deals with the following aspects of LIBS and its application: (i) the fundamentals of this analytical measurement method, (ii) the matrix effect and its influence on the LIBS analyses of various biological tissues, (iii) the role of signal enhancement methodologies and artificial intelligence models to advance the method for analyses of biological sample materials, and (iv) applications of LIBS in cancer and calcified tissues investigations. This article also addresses challenges and opportunities encountered in these applications and discusses prospects, providing a comprehensive overview of the current state and potential advancement in LIBS technology.

## 1. Introduction

LIBS has garnered considerable attention from researchers in biomedical fields over recent years, yielding prolific outcomes either alone or in combination with other supporting techniques [1,2,3]. This laser-induced plasma spectroscopy method stands out for its speed, non-destructiveness, real-time analysis, minimal or no sample preparation requirement, multi-element detection, and relatively inexpensive instrumentation [4]. It has been effectively used for environmental monitoring [5,6], agricultural materials inspection [7], archaeological investigation [2,8], geological mining [9,10], industrial applications [4,11], space exploration [12], forensic sciences [13], health sciences [14,15], and biological detections [16,17].

Cancer remains the leading cause of death globally, mainly due to its silent onset and lack of early symptoms [18,19,20]. Despite advances in bioscience, early detection—a critical factor for successful treatment—remains a significant challenge. Cancer diagnosis today relies on conventional biopsies, imaging [computed tomography (CT)/magnetic resonance imaging (MRI)/X-ray/positron emission tomography (PET)/mammography (MMG)], and laboratory tests [21,22,23,24], which are effective but often slow, expensive, and invasive [21,25]. Alternatively, optical analytical techniques [atomic absorption spectroscopy (AAS) [26], laser-induced breakdown spectroscopy (LIBS) [27], Raman spectroscopy (RS) [28], inductively coupled plasma mass spectrometry (ICP-MS) [29], and X-ray fluorescence (XRF) [30] offer rapid, cost-effective, and less invasive diagnostic efficacy. Among others, LIBS is considered an emerging state-of-the-art tool with significant advancements in understanding cancer biology [31,32].

Calcified tissues serve as elemental archives due to hydroxyapatite’s (HA) affinity for toxic metals and metabolic markers [33]. Teeth disorders and metabolic bone diseases alter HA crystallography, changing crystal morphology, orientation, and alignment with collagen, as well as elemental composition, in a manner distinct from that found in normal calcified tissue [34]. Their composition is analysed by two methods: dissolution, which dissolves samples and hinders in vivo research, and spectroscopy, which is semi-destructive and provides precise spatial mapping, enabling more accurate in vivo and in vitro analysis than the former [16].

**Figure 1:**
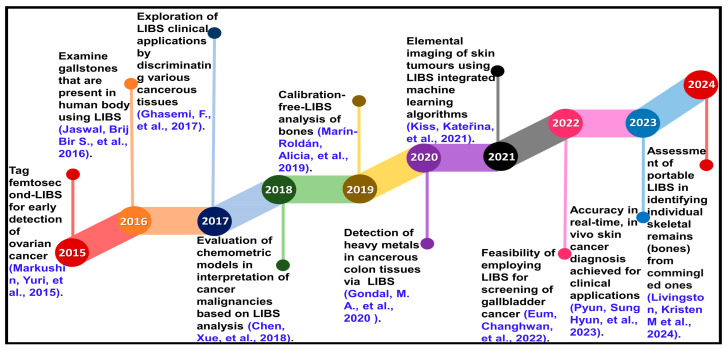
LIBS timeline highlights the achieved milestones in the field of cancer detection and calcified tissue analysis over the past decade [39,45,46,47,48,49,50,51,52,53].

LIBS enable rapid elemental ana lysis of cancerous and calcified tissues, serving as a powerful tool for pathological diagnosis and physiological monitoring [35,36,37]. Classification of pathologies corresponds to variations in laser-induced plasma characteristics, elemental concentrations, detection of toxic metals, molecular decomposition, de/remineralisation, chemical imbalances, calcification, and carbonisation [34,38]. LIBS achieved significant milestones in cancer diagnostics and the investigation of calcified tissue, as highlighted in Figure 1. However, its clinical adoption remains limited due to challenges, for instance the matrix effect, issues in signal reproducibility, sensitivity issues from tissue heterogeneity, and ablation-induced sample destruction [39,40,41]. The path forward lies in intelligent calibration systems and hybrid analytical approaches that compensate for these technical challenges by providing linearity, sensitivity, and robustness to signals [42]. In 2019, Gaudiuso et al. [43] devoted review sections to LIBS-based analysis of calcified tissues and the application of LIBS for cancer diagnosis using fluid specimens of humans and animals. In 2022, Khan M.N. et al. [40] reviewed advancements in LIBS for diagnosing various cancers using different tissue samples and body fluids and Khan Z. et al. [44] reviewed trace element detection in biomaterials and other materials. The importance of LIBS in calcified tissue analysis was highlighted by Singh et al. [16] a decade ago.

The lack of availability of LIBS-oriented research and its unexplored avenues in cancer diagnosis, calcified tissue analysis, and the effect of cancer on calcified tissues urge us to write this article to attract, convince, and engage researchers and readers toward med-LIBS. The primary goal of writing the present article is to keep readers updated on the latest ongoing research trends (in med-LIBS) by covering literature (from 2015 to 2025) related to LIBS application in cancer diagnosis and calcified tissue examination. This work provides a brief overview of LIBS, including the setbacks associated with the matrix effect, the methods used for signal enhancement (SE), and the artificial intelligence (AI) models applied for spectral analysis. It includes a critical evaluation of LIBS as a diagnostic tool in multiple types of cancers: skin, breast, blood, lungs, stomach, colon, ovarian, gallbladder, oral, cervical, and brain. Furthermore, an overview is presented on published research work on human and animal calcified tissues (teeth, bones, kidney stones, and chicken shells) from LIBS-medical aspects. Finally, before concluding, the article discusses shortcomings, potential solutions, and prospects.

## 2. Fundamentals of LIBS and Elemental Composition of Human Body

**Figure 2:**
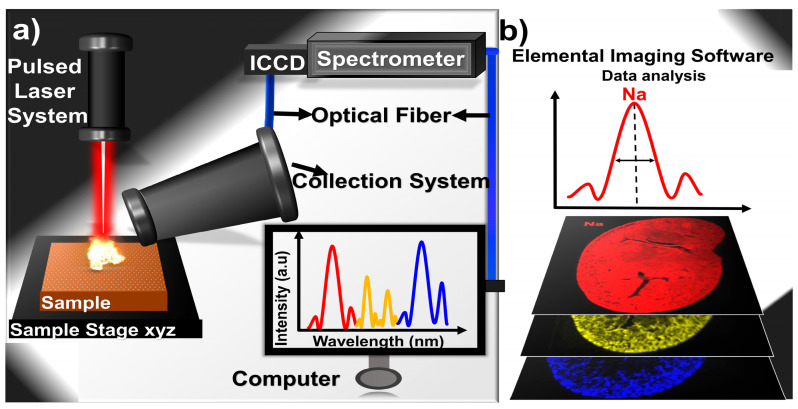
(**a**) schematic diagram of LIBS instrument showing primary components: pulsed laser used to generate plasma of sample placed in moveable sample stage, optical collection system connected to spectrometer by an optical fibre, and computer system for qualitative and quantitative elemental analysis; (**b**) spectral analysis by homemade elemental imaging software built in LabVIEW environment (adapted from [38] with permission).

LIBS is a spectro-analytical technique in which a laser pulse ablates a small volume of material from the sample surface, creating a microplasma. As the plasma cools, it emits characteristic wavelengths of light that act like a fingerprint for the elements it contains (Figure 2a).

The spectrometer records these emissions to get the spectrum. These spectra were further analysed to determine the elemental composition of the target [47], and the images formed are shown in Figure 2b. LIBS fundamental principle can be summarised in four steps: (i) laser ablation process, (ii) plasma formation and plasma plume expansion, (iii) plasma emission, and (iv) spectral analysis [54,55,56].

A basis to advance understanding of LIBS can be developed going through these aspects: fundamentals (laser parameters, specimen properties, and plasma chemistry govern the complex interaction processes) [54], instrumentations and methodologies [34,56,57,58,59,60], pulsed laser–tissue ablation [61], plasma formation and plasma diagnostics [56,60,62,63,64,65], spectral analysis [54,55,56], advance data processing (AI-based machine learning ML models) [66,67], and elemental imaging [68,69]. Laser–matter interaction, various forms of ablated material and plasma interaction, and laser–plasma plume interaction (caused by plasma shielding) are underlying mechanisms for understanding LIBS analytical outcomes [62]. Due to the high complexity of interaction phenomena, specific theoretical models exist, and the most commonly used for plasma modelling is local thermodynamic equilibrium (LTE) [65]. There are two primary LIBS formalisms adopted for the elemental quantification of specimens: calibration curve (CC-LIBS) and calibration-free (CF-LIBS). In CC-LIBS, standard reference materials are required to quantify the elemental content of the specimen by plotting a calibration curve. In contrast, the CF-LIBS approach is employed to determine the elemental concentration of diverse samples without standard references or matrix-matched materials, eliminating the need for a calibration curve [70,71,72,73].

In nanosecond (ns) pulsed laser ablation, plasma is formed during laser pulse, and the trailing part of the pulse interacts with plasma, leading to plasma reheating and persistence. The plasma formation time depends on the laser pulse length because the pulse duration determines how long energy is delivered to the target materials, thereby impacting the mechanisms and time scales of plasma formation. For ns laser pulses, it is an order of a few ns to tens of ns, whereas for fs laser pulses, it is an order of 1 ps [74]. Ultra-short laser pulses with durations ranging from picoseconds (1 ps = 10^−12^ s) to femtoseconds (1 fs = 10^−15^ s) are commonly used [75]. The fs pulse plasma evolves faster, with a lifetime of several hundred ns, depending on laser fluence and various other factors. In contrast, ns plasma evolves relatively slowly and has a longer lifetime of µs [74,75]. In femtosecond pulsed laser ablation, plasma is formed after the laser pulse, so interaction between plasma and laser can be avoided. Due to the absence of plasma–laser interaction and the very short thermal penetration length for fs pulses the absorption of laser energy is localised to the irradiated area and the heat-affected zone (HAZ) is strongly reduced compared to ns pulses [76,77].

Fs laser pulses offer high ablation efficiency as the ablation thresholds are lower than with ns pulses. Fs laser ablation depth of 6 µm on a thin tissue section of liver metastases (from a colorectal cancer (CRC) patient) was reported, allowing fast in-depth multi-elemental profiling at cellular spatial resolution [78]. A maximum ablation rate of 0.66 mm^3^/s in porcine femur for laser pulses with wavelength of 515 nm and repetition rate of 250 kHz is reported in [79]. These pulses promoted less selective ablation and reduced dependence on the material matrix, which favours determination of elements in dental tissue (dentine) [80]. Fs LIBS offers reproducible spectra in the pathological tissues (section of liver metastases of CRC patient, breast tissues with tumour, and lymph node with metastasis breast cancer) at lower energies due to less interaction between fs pulses and plasma plumes [81]. Fs-LIBS elemental imaging of melanoma tumour tissue (skin cancer) provides a spatial resolution of 15 µm [82]. It is also used as a real-time feedback control system to ensure the removal of bone tumours, thereby reducing the need of repeat surgeries [83]. Their ability to reduce damage to dental tissues and surrounding nerves from undesirable thermal destruction enables the use of a feedback loop in which the clinician can both diagnose and remove carious lesions [84].

Elemental concentration in the human body can vary for several reasons, primarily related to physiological and quality of living standards [85], including age [86], gender [86,87], diet [88], habitat [89], and environmental exposure [90]. Another source of elemental variations can be disease [91]; among many, our prioritised content focused on various forms of cancer diseases and pathological calcified tissue abnormalities, and researchers identified elemental biomarkers based on LIBS data collection. It is worth noting that the interpretation of LIBS data has a significant impact on determining relevance from a biological perspective [92]. Even for the same physiological or pathological conditions, elemental variation may not be unique. LIBS investigators have not succeeded in defining particular criteria for certain types of disease diagnosis, and novel research still requires confirmation. Additionally, spectro-analytical outcomes highly depend on experimental instruments and conditions, the nature of tissues, plasma dynamics, approximations for spectral analysis, formalisms for quantification, statistical methods, and the use of AI models for characterising biological tissues [85,93].

**Figure 3:**
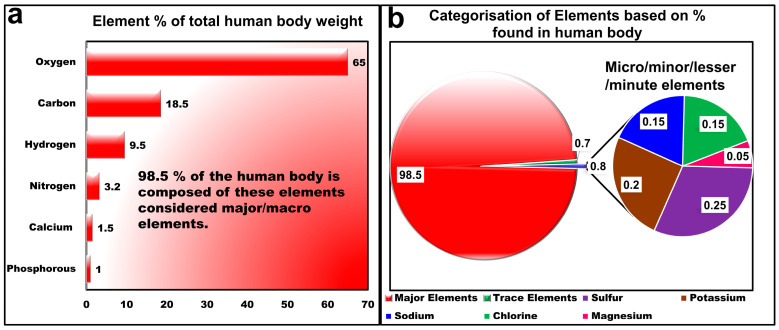
(**a**) Most abundant element in the human body; (**b**) categorisation of elements into major, minor, and trace elements based on percentage in the human body (data collection [94]).

Major/macro, micro/minor/lesser/minute, and trace elements constitute 98.5%, 0.7%, and 0.8% of total human body weight, respectively, as illustrated below in Figure 3 [94]. Macroelements are crucial for regulating glucose metabolism and detoxifying contaminants, whereas micronutrients are essential for the body in minute quantities for the proper growth and development of organs. Trace elements, though scant, are pivotal: their imbalance triggers the disease, from hyperglycemia to toxic metal poisoning, serving as silent biomarkers for cancer and calcified tissue disorders [68,91,92]. Yet quantifying them remains a challenge that one LIBS could overcome [92] if its limitations, particularly matrix effects, are addressed.

### 2.1. Matrix Effect

The matrix effect is the influence of a specimen’s chemical composition and physical properties on analytical signals, causing variations in emission line intensities and errors in analyte quantification [95]. The measured intensity of an analyte chemical element may show a dependence on the concentration of another element in the sample material, for instance (cross-sensitivity). Also, the spectrochemical analyte signal may depend on the sample material’s condition, e.g., its hardness, mass density, microstructure, and water content, even if the concentration of the analyte is independent of such conditions. Laser ablation of tissue is initiated by the absorption of laser radiation in the surface of the sample material and, in case of short ns pulses, in the early plasma induced by the trailing edge of the pulse. The penetration depth of absorbed laser pulse energy into the sample and, therefore, the amount of ablated material per pulse, the energy of ablated species in the plasma, and the composition of the plasma plume are dependent on the condition/state of the tissue investigated. The measured intensity of continuous radiation which masks relevant elemental information [62,96] and, most importantly, the intensity of specific analyte emission lines and bands at later stages of plasma expansion are therefore dependent on such conditions. The matrix effect has direct involvement in LIBS signals and cannot be completely eliminated; however, its adverse effects can be reduced to improve LIBS quantifications [97]. This unwanted phenomenon can cause (i) reduction in signal sensitivity and detection limit due to variations in plasma properties [98], (ii) non-linearity in calibration curves due to non-uniform response of the LIBS measurement system to the concentration of analytes across different matrices [99], (iii) non-uniform ablation due to different physical and chemical properties of matrices [100], and (iv) elemental fractionation due to non-stoichiometric ablation [101].

In LIBS (and also for other laser ablation based spectrochemical techniques) the matrix effect in soft and hard tissue is very significant. For cancer diagnostics soft tissues are typically characterised. The effect primarily originates from organic and inorganic content of tissues which have distinct elemental compositions that, in turn, influence their plasma parameters [49,102]. Research [103] has demonstrated that plasma temperature and electron number density are inversely proportional to carbon content in samples, which is associated with ablation rates and the recombination process within the plasma. Laser-induced plasma of organic tissues is typically dense, with several optically thick spectral lines, resulting in a pronounced background that can obscure the subtle analytical signals. These organic tissues contain water which acts as a thermal buffer, reducing ablation efficiency and plasma temperature. Consequently, this leads to signal suppression and poor reproducibility. Protein and lipid increase molecular emission (CN and C_2_) due to their high carbon content which can interfere with metal emissions. Accurate quantification of metallic trace elements is essential for assessing the physio-pathological states of tissues. Relatively, soft tissues exhibit weaker mineral emission making the detection of trace elements more challenging [104]. Due to the matrix effect, poor sensitivity and a high limit of detection (LOD) are hurdles in Tag-LIBS readout for the accurate quantification of human serum albumin (HSA) [105], multiple tissues, tooth-bone, are encountered in the same specimen. Their examination is challenging [106], and matrix/non-matrix elements of dental tissues (healthy or carious) and filling material (amalgam) behave differently at varying laser fluences and pulse numbers [107]. Approaches to reduce the undesirable matrix effect in LIBS include the following: (i) adoption of suitable sample preparation methods (e.g., with pressed samples plasma temperature fluctuation can be reduced [103]); (ii) optimisation of experimental parameters (e.g., laser beam defocusing and spectrometer delay [99], use of ultraviolet laser wavelength (266 nm) for higher ablation efficiency, improved plasma stability, and sharper spectral lines in calcified matrices [108]); and (iii) selection of data processing methods (e.g., matrix-matched external calibration [109,110], automated matrix recognition for identification of different tissues in a sample using ML algorithm improved elemental quantification [109], image fusion techniques extracting matrix independent information of bones from LIBS data profiling [111]). Additionally, various matrix-matched reference materials are available to enhance the accuracy and precision of quantitative analyses. Several calibration materials and methods are used for the trace elemental quantification of soft tissues (such as brain, liver, and hair) and hard tissues (teeth and bones) via LIBS and related techniques such as LA-ICP-MS [110]. The optical diagnostic approach of front-face fluorescence (FFF), used as a complementary method, can reduce the reliance of LIBS elemental results on the tooth matrix for early detection of pathologies [112].

Despite employing multiple strategies, researchers have not reached a consensus on which method is most suitable to reduce the matrix effect for different types of tissue. An overview on the reported influence of the biological matrix on the LIBS analytical performance of tissues is given in Table 1. The heterogeneous nature of samples contributes to this effect. LIBS analyses of tissues with similar elemental compositions (e.g., fat and nerves) but different structure (matrix) require different approaches for analyte quantification. Results from ex vivo analysis cannot be reliable transferred to in vivo applications, where the tissues are immersed in blood and other body fluids and surrounded by other organs [113]. A universal procedure to correct LIBS spectra and data for this effect is lacking due to complexity and diversity of biological tissues. Few efforts have been made to mitigate the matrix effect on LIBS performance using animal tissues [49,96,109,113,114,115]. Studies on human tissues [116] are lacking, which may be due to ethical issues and the unavailability of human tissues.

### 2.2. LIBS Signal Enhancement Strategies

LIBS is encountering numerous difficulties (mentioned in Table 1) in examining biological tissues. Several methods have been tried to address these issues. Among many methodologies, nanoparticles enhanced (NE-LIBS) is recommended due to its potential for on-site analysis (in vivo studies) of biological tissue. But in this method, enhancement of emission signals depends on the size, shape, distribution, and concentration of nanoparticles (NPs), which are not easily controlled. Colloidal silver nanoparticles (Ag-NPs) can enhance the LIBS spectral intensity of metals in serum samples deposited on filtration paper by mitigating the coffee ring effect [126]. The reported enhancement factors for Ca, Mg, and K are 1.76, 1.85, and 3.10, respectively [127]. Sprinkling of bio-synthesised Ag-NPs on bovine bones before analysis enhanced the sensitivity of LIBS signal [128]. Ag-NPs on antibodies enhanced the detection of europium (Eu) and ytterbium (Yb) up to 12 times [129]. The formation of zinc oxide nanoparticles in acid (eugenol) for teeth analysis [130] poses health issues for in vivo examination. Its limitation is that it can only improve the sensitivity and LOD of elements, yielding linear calibration curves.

Tag-LIBS readout sensitivity for human serum albumin (HSA) detection is improved by using upconversion nanoparticles (UCNPs), collinear DP-LIBS configuration, and a modified optical collection system. The reported LOD of TAG-LIBS for HSA is 0.29 ng/mL which is comparable to 0.37 ng/mL when measured with a standard enzyme-linked immunosorbent assay (ELISA). However, the performance of Tag-LIBS is still significantly lower than that of the gold standard readout, the upconversion-linked immunosorbent assay (ULISA) (LOD: 0.17 ng/mL). Additionally, for Tag-LIBS the signal-to-background ratio (SBR) is 3.5 times lower than that of ELISA, and the results demand validation on multiple biomedical specimens [105]. NETag-LIBS, a combination of NE-LIBS and Tag-LIBS, has enhanced sensitivity in the detection of biomarkers when an optimum concentrations (0.05–0.1) mg/mL of Ag-NPs is used [129].

Spark discharge (SD)/spark-assisted (SA) LIBS methods have been employed to discriminate between healthy and cancerous gastric tissues [123] and healthy (infiltrate) and brain tumour tissues [131] based on emission signals of Ca and Mg. However, emission enhancement is not significant enough, and signal enhancement factors are not reported, which may be due to experimental uncertainties. In surface-enhanced (SE-LIBS) superhydrophobic substrates, polydimethylsiloxane (PDMS), can elevate the detection of trace elements in the blood serum of an oral cancer patient, improving sensitivity and specificity to 88% and 96%, respectively [132].

Fs LIBS enables one to map the distribution of tooth elements with spatial resolution of 100 µm [80]. Fs-DP-LIBS signals are enhanced up to 5-fold compared to fs-SP-LIBS when exploited to bovine tissues (liver and muscle). However, the enhancement factor is depending on several factors including physicochemical matrix effects, sample preparation, environmental conditions, and instrument specifications [133]. Fs-pulses enable the removal of caries from dental tissues through a precise ablation process, but optimisation of the fs-LIBS experimental setup is the prerequisite for controlling temperature [84].

Very few studies have exploited dual/double pulse (DP-LIBS) for biological specimens. One is for bovine tissue analysis and the reported signals are 5-fold those of SP-LIBS [133]. The other is about HSA where a collinear DP-LIBS configuration is employed [105]. Although the potential of DP-LIBS, electric field-assisted (EF-LIBS), and microwave-assisted (MW-LIBS) has been demonstrated in other scientific domains [134,135,136,137,138,139,140], their specific advantages and limitations in the analysis of medical samples remain unexplored and require systematic investigation.

### 2.3. Artificial Intelligence in LIBS

LIBS-integrated ML and DL algorithms offer advantages in large and complex spectral data processing, including pattern recognition for rapid classification, spectral feature extraction, and high precision and accuracy by mitigating sample matrix effects and compensating for self-absorption (SA) interference [141]. Generally, model training requires complex data pre-processing to filter signals using various approaches including baseline correction, normalisation, and outlier detection [142]. Nowadays, the spectroscopic output contains thousands of variables and millions of spectra which are impossible to process manually, but AI algorithms can efficiently and effectively accomplish such complex processing [143]. Although these models have several advantages, for data from biological samples they are encountering many challenges (overfitting, underfitting, optimisation and validation losses, inconsistency, absence of physical embodiment, lack of interpretability, and probabilistic outcomes) [144].

Principal component analysis (PCA) is an unsupervised ML algorithm. It is commonly applied for dimensionality reduction of LIBS spectra to enhance model robustness for the diagnosis of oncological diseases [31,48,119,122,132,145] and the analysis of hard tissues [83,96,112,146,147,148,149]. For diagnosis of human blood malignancies (lymphoma and myeloma) the measured spectral data of order 4800 (number of spectra) × 24 (number of spectral lines) was reduced to 4684 × 12 [48], for discrimination of lymphoma the original data set 3240 × 16 was minimised to 3240 × 6 [119], and for classification of tooth tissues the data matrix 5589 × 9 was compressed to 5589 × 2 [149]. After data reduction through PCA, the average sensitivity of the supervised ML algorithms linear discriminant analysis (LDA), quadratic discriminant analysis (QDA), and kernel nearest neighbour (KNN) for serum samples (of blood cancer patients and healthy subjects) was 82%, 92%, and 96%, respectively [48]. LDA accuracy for discrimination of lymphoma was 99.78%. Validation losses and misclassification cases, even for a small dataset of 17 patients, remain a significant concern for the algorithm’s performance [119].

Artificial neural network (ANN)-supervised ML models are frequently used in the classification of LIBS spectral data for cancer [27,81,118,122,150,151] and calcified [147,152] tissues with an accuracy of over 89%. The major concern is that none of these studies [27,81,118,122,147,150,151,152] reported all the required model parameters: architecture (number of hidden layers, number of neurons per layer, hidden activation function, and output activation function), learning (learning rate, batch size, and loss function), and regularisation (early stopping, dropout rate, and L2 regularisation rate). It is necessary to incorporate such information in published research work; without it, these studies cannot be reproduced. Consequently, the results remain specific to the application of a particular lab. In addition, many other ML models (PLS-DA [118,152], logistics regression (LR) [147,153], support vector machine (SVM) [31,154,155,156,157,158], random forest (RF) [81], boosting tree [159], bagged tree [160], bagging voting fusion (BVF) [158], and frameworks (XGBoost [121] and AdaBoost [150])) are used in both of the mentioned medical fields. However, the limitations of ML models and frameworks prevent LIBS technology from being applied outside of laboratories. To overcome such issues, data scientists built a deep learning architecture and introduced reinforcement learning.

Deep neural network (DNN) and convolutional neural network (CNN) are deep learning algorithms designed for tabular/structural and imaging/visual data, respectively [144]. Despite the restriction on using CNN on imaging data, a few researchers apply them to sequential data [121,161,162,163]. They claimed classification accuracy of 97.72% for various cancer cells (cervical, liver, and colorectal) [161], 82% for breast cancer identification from healthy tissues [162], and 92% classification sensitivity (first batch) for breast cancer [163], and 93% for lung cancer staging [121]. Biases and small perturbations intentionally introduced in the input data raised questions about the credibility of this research. In addition, the unavailability of architectural and model parameters in several works, (i) DNN for skin cancer diagnosis [52], (ii) RF-1D ResNet for lung tumour identification [164], and (iii) GRAN for breast cancer [163], does not support their adoption in the medical field, because these frameworks inherit certain limitations (substantial amount of training data, unclear working mechanisms, requirement of high computation power, environmental impact, security vulnerability, and poor generalisation) [144]. Table 2 highlights some shortcomings of integrating AI models for the interpretation of LIBS data from oncology and calcified tissue research, along with possible solutions and suggestions.

## 3. Implementation of LIBS in Oncology

The first research on cancer via LIBS was performed in 2004, focusing on discriminating cancerous tissues from healthy ones (in dogs) [167]. This foundational research work laid the groundwork for subsequent studies examining LIBS in multiple cancer diagnostics. LIBS cancer screening and pathology identification efficacy provides simpler and more realistic objectives as a complementary approach to traditional histopathological examination [117].

Several methodologies are employed to investigate LIBS as a diagnostic tool in pathological examinations, including (I) classical LIBS approach [168], which involves comparing spectral features, plasma parameters, and elemental composition of cancerous and healthy tissues, and validated against traditional analytical methods. This typical method has been deployed to investigate multiple types of cancers: skin cancer [117], breast cancer [35,47], stomach cancer [123], colon cancer [47,50,120], brain cancer [131], bone invasive oral cancer [153], and gallbladder cancer [39]. (II) LIBS elemental imaging, where spatial resolution is a critical parameter, as it governs the ability to distinguish fine structural and compositional features within a sample. It is crucial for cancer diagnosis, where detailed elemental mapping can reveal subtle tissue changes. The technique involves scanning the sample with micrometre-scale laser pulses in a raster pattern. Each pulse ablates a small area, producing a LIBS spectrum that reflects the elemental composition at that point.

Advanced spectral analysis quantifies elements of interest, typically biomarkers, and generates high-resolution 2D or 3D maps that visually represent their spatial distribution [27,47,169,170]. This methodology is used in the identification of skin cancer [125,171] and malignant pleural mesothelioma (MPM) cancer [29]. (III) AI-assisted LIBS (supervised machine learning methods [168]) focuses on spectral features related to cancer markers and matches spectral peaks to identify elements associated with pathologies. Spectral features (peak intensity, area under the curve, and ratio between elements) are extracted, such as to use them as inputs for ML model. The steps are as follows: (i) training of model (using 70% known data set (training set)) to learn the relationship between spectral feature and cancer classification, (ii) optimisation of model (using 15% known data set (validation set)) by controlling hyperparameters (to improve model accuracy and prevent overfitting) to attain optimum classification performance, and (iii) testing and evaluating the model performance (using 15% unseen data set (test set)) using accuracy, sensitivity, specificity, and receiver operating characteristic-area under curve (ROC-AUC) to measure effectiveness of model in diagnosing cancer. This methodology has been used in several cancer studies, including skin cancer [52,119,145,150,168], breast cancer [81,162], lung cancer [159,160,164], ovarian cancer [27,172], colon cancer [81,161], cervical cancer [31,161], blood cancer [166], thyroid cancer [154], and brain tumours [151,165]. (IV) LIBS elemental imaging and machine learning, in which methods II and III are combined for screening, diagnosing, and monitoring of cancer, has been employed for skin cancer [51,82]. In addition, other strategies are also incorporated with LIBS to enhance cancer diagnostic and treatment efficacy, signal enhancement methodologies (surface-enhanced (SEN-LIBS) [132], and spark-assisted LIBS [123,131]), Tag-LIBS [45,173], NE-LIBS [129], ultrashort LIBS [81], and hybrid technology [35,121,171,174]. Before delving into each cancer type, it is essential to have basic information on LIBS instrumental and experimental parameters used in various oncological investigations, as presented in Table 3.

### 3.1. Skin Cancer

Skin cancer leads global cancer statistics, with over 1.5 million cases reported in 2022. Melanoma, the most lethal form, shows higher prevalence in white populations and males. Non-melanoma types, such as basal cell carcinoma (BCC), squamous cell carcinoma (SCC), and Merkel cell carcinoma (MCC), further add to substantial diagnostic load [176,177]. Multiple non-invasive optical modalities, including dermoscopy, multiphoton microscopy, confocal microscopy, dermatofluoroscopy (DMF), and optical coherence tomography (OCT), offer real-time imaging for early detection [19,21,23,24,178,179,180]. These techniques face critical limitations, including poor penetration depth, low resolution, and an inability to distinguish between ambiguous lesions. Consequently, they are insufficient as standalone diagnostic tools and continue to rely on histopathological confirmation via biopsy. Optical imaging remains a valuable adjunct, but not a replacement, for more definitive microscopic and spectroscopic analyses [21]. In this context, LIBS combined with optical imaging methods can be an excellent choice for addressing skin diseases clinically, where pathologists can effectively use it to screen, diagnose, and perform therapeutic operations before making additional treatment recommendations [25].

Khan and his co-workers (2020) [118] proposed a stage-wise classification of melanoma skin cancer using ML algorithms. The identification of melanoma in skin tissues through multiple classification models and the declared achievement of classification accuracy (100%). Pyun S.H. et al. (2023) [52] used LIBS technology in real-time skin cancer detection. The DNN model is trained and tested based on the LIBS-identified biomarkers during in vivo analysis. They achieved sensitivity (94.6%) and specificity (88.9%) for diagnosing skin malignancies while preserving tissue integrity at both microscopic and macroscopic levels. Concluded that LIBS is an attractive candidate for skin treatment and has real potential in the field of dermatology. However, the primary concern is that these models have not been evaluated on real patient data (from hospitals) and have not been consulted with practitioners to determine whether the classifiers are specific to such cancers or originate from other sources. These models are predictive and unable to provide causal explanations. To date, LIBS-ML studies have not incorporated demographic, medical history, social activity, genetic, and environmental factors of patients and participants into the training and testing of algorithms. Causal inference models have the potential to incorporate the mentioned factors and play a pivotal role in understanding disease propagation [181].

**Figure 4:**
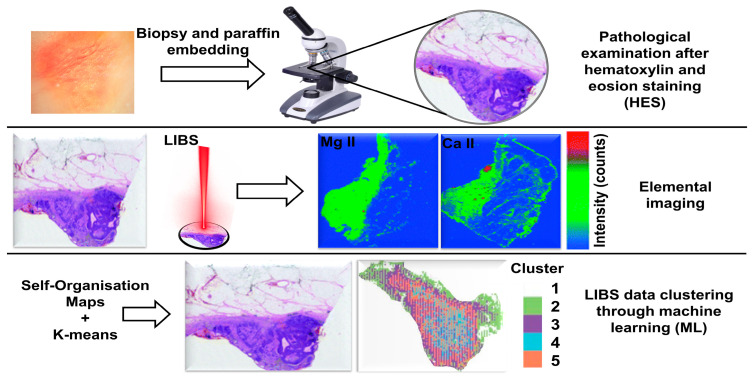
Histological [hematoxylin and erosion staining (HES)] images complemented with LIBS elemental images of Mg and Ca for basal cell carcinoma (BCC) tumour sample, adapted from [51] with permission, licensed under CC by 3.0.

Kiss et al. (2021) [51] conducted a spectral study, and the analysis of four biogenic elements (Mg, Ca, Na, and K) showed higher concentrations of Mg, Na, and K in tumour-affected tissues. The progression of the tumour was examined by capturing biotic elemental (Mg, Na, and K) imaging. It was correlated with the concentration and spatial distribution of elements within malignant tissues, as illustrated in Figure 4. The distribution and proliferation of calcium are not well understood outside the tumour region. The authors declared the novelty of their work in hyperspectral data processing and k-clustering analysis for discriminating cutaneous tissue from its healthy surroundings. In research work, LIBS-3D imaging involves a complex sample preparation procedure, such as FFPE, which contrasts with the fact that LIBS typically require minimal or no sample preparation. An experimental method is lengthy, as it involves background correction for every single laser shot. The destructive effect on tissue, spatial resolution, and self-organising map (SOM) data interpretation information is not reported for in-depth understanding. Choi et al. (2021) [82] performed ultraviolet (UV-fs-LIBS) imaging of melanoma-affected skin tissue to classify melanoma and dermis. During the experiment, femtosecond laser operated at the UV region (343 nm) with a laser energy of 50 µJ provided a consistent crater pattern with minimal debris on the periphery of the exposed tissues. Spectral analysis revealed the intensity ratio $\left(I_{K}/I_{\mathrm{CN}}\right)$ and peak intensity $\left(I_{\mathrm{CN}}\right)$ of the element as a classifier to identify melanoma regions. LIBS imaging was performed, and results were validated with hematoxylin and eosin (H&E) staining images, confirming the identification of cancer with LIBS. They suggested that a UV-fs-laser is more appropriate for imaging, and achieved a spatial resolution of 15 µm.

In the future, it could be a useful alternative or complementary tool for melanoma diagnosis. In this work, as in any other research, data fairness remains a concern, as biases in data (such as selection bias, social bias, and sample bias) can impact the model’s performance. Table 4 compares the LIBS research studies with the considered gold standard optical and spectroscopic methods applied in skin cancer diagnosis.

### 3.2. Breast Cancer

Breast cancer affects more women than any other cancer and has the highest mortality rate among female cancers. In 2020, an estimated 1,233,465 breast cancer cases were recorded worldwide among females aged 15–59 years, according to WHO statistics [189]. They reported a higher incidence rate in developed countries. However, the availability of advanced medical equipment helped them to fight against such diseases, which resulted in low casualties. Conversely, fewer cases are recorded by governing bodies in developing countries, but their survival rates are significantly lower due to a lack of awareness, inadequate medical resources, and high treatment costs [190,191]. Contemporary methods such as MRI, ultrasound, thermography, biopsy, and tissue sampling are widely used for screening breast cancer [18,22,192]. LIBS was used to study breast cancer in 2012 by quantifying the concentration of trace elements [155]. Idrees et al. (2023) [175] compare the effect of sample selection (whole blood and serum) on the diagnostic accuracy of LIBS-ML data interpretation. Accordingly, the choice of whole blood samples yields a lower error rate and might assist in early cancer screening. The research suggests LIBS cancer diagnosis may depend on the state and quantity of blood samples, but the point needs further confirmation.

Ghasemi et al. (2016, 2017) [35,47] used the classical LIBS approach to discriminate cancerous (breast, larynx, tongue, and colon) tissue from normal ones, but the measured plasma parameters and trace elemental ratios of malignant and benign tissues are very close to each other, and higher and intense for abnormal ones. The minor error in measuring plasma temperature propagates to cause high uncertainty in determining other parameters, electron density, and plasma frequency, which ultimately affect the accuracy of elemental quantification. The approach demands cross-validation, which is an extra effort and time-consuming. Table 5 lists several LIBS breast diagnostic investigation and their comparison with other state-of-the-art technologies that have evolved over the last 11 years.

### 3.3. Colon Cancer

**Figure 5:**
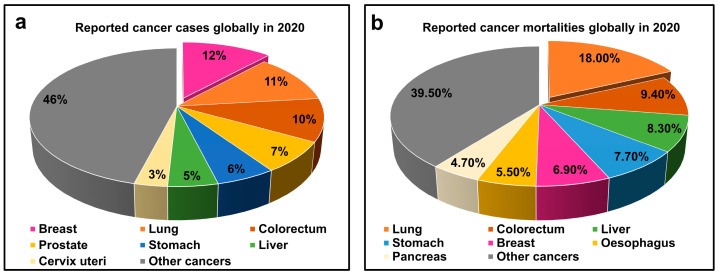
Reported distinct cancer cases globally in 2020: (**a**) incident rate; (**b**) casualties [189].

Colon/colorectal cancer is the third most common cancer in terms of morbidity rate and the second leading cause of cancer-related mortalities worldwide, as statistically shown in Figure 5. Gondal et al. (2020) [50] determined the elemental concentration of heavy toxic metals in both normal and affected colon tissues using two distinct techniques (LIBS and ICP-OES). Researchers analysed cancerous colon tissue and correlated with accumulated heavy metals (Hg, Cr, Pb) using CF-LIBS. They compared the results and found that no toxic metals were detected in healthier tissue. In contrast, the presence of highly toxic metals (Hg, Cr, Pb) in cancerous colon tissues indicates tumour growth. The use of small sample sizes (n = 15), the lack of statistical analysis, and the absence of a follow-up study at a larger scale restrict the generalisation of the results and prevent confirmation of the conclusion.

### 3.4. Stomach Cancer

Globally, gastric/stomach cancer accounts for the fifth-highest cancer incidence. Its incidence has experienced a significant decline over the last five decades. Unfortunately, it remains a serious health concern as the third most important cause of cancer-related global deaths [197]. Spark discharge-assisted LIBS (SD-LIBS) was employed to investigate the feasibility of differentiating malignant tissues from normal stomach tissues for diagnosing gastric cancer. Neoplastic (malignant) and non-neoplastic (normal) stomach tissues were identified based on their respective distinct atomic emission spectra and measured elemental concentration. It was found that the Ca and Mg content is higher in cancerous tissues than in normal tissues for the same individual. However, only five patients participated in this research activity, which was insufficient to reach a firm conclusion. Findings are neither statistically nor medically validated and have not been confirmed using traditional elemental quantification methodologies [123].

### 3.5. Lung Cancer

Lung cancer has been a serious public health concern due to its high incidence and mortality rate. Globally, the lung cancer death rate in 2020 was the highest among all types of cancers, as shown in Figure 5. Approximately 1.8 million people are diagnosed with this disease, and 1.6 million die annually; these statistics are relatively higher than other oncology-related threats [20,198]. Lin et al. [159] differentiated between lung tumour tissue and boundary tissue by using LIBS-ML models. After pre-processing the LIBS spectral data of 90 tissues from 45 patients, principal component analysis (PCA) is used for dimensionality reduction, and random forest (RF) is used for feature selection. Limitations of PCA and RF (variance may not be informative, biasing towards high cardinality features, and inability to provide causal interpretation) may cause the loss of important information required for diagnosis. PCA-SVM and RF-BT models are trained and tested with compromised features. RF-BT diagnostic performance is declared to be superior to that of other models. However, the difference among model index indicators is less than 6% for all models. Li et al. (2024) [158] developed a bagging voting fusion (BVF) algorithm and proposed it as a method to overcome the limitations of single models in identifying complex cancers. In BVF, five models (SVM, ANN, KNN, QDA, and RF) were fused at both training and decision levels to process LIBS data to increase the diagnostic accuracy of multiple types of cancers (liver, lungs, and esophageal). The model claimed to achieve an accuracy of ~92% and a recall of ~93% for all serum samples, outperforming the best single model (SVM: accuracy of ~76%, recall of ~78%). To conclude, LIBS-BVF enables rapid (<3-min) and precise detection of a multitude of cancers, representing a transformative approach to clinical cancer diagnostics. In the above studies, dimension reduction approaches (PCA, FS, FE) are used to enhance model performance by sacrificing some of the information that must be given as input to build them. The high accuracy of LIBS-ML algorithms does not guarantee a reliable cancer diagnosis, as some ML models lack causal explanation mechanisms, which complicates the evaluation of their scientific validity and undermines the trust of oncologists [181].

### 3.6. Cancer Studies in Animal Samples

Han et al. (2016) [145] explored the feasibility of using LIBS to distinguish melanoma lesions from surrounding dermal tissue. They conducted elemental analysis on optimised pellet samples from melanoma-implanted mice. However, the limited sample size (n = 10, 1470 spectra) prevents the drawing of robust statistical conclusions, and the use of homogenised samples raises questions about clinical relevance. Most crucially, the identified biomarkers require validation in actual human melanoma cases. Further, this work lights the path forward but underscores the need for larger and more physiologically human-relevant studies. Zhao et al. (2024) [150] employed adaptive boosting (Ada-Boost) combined with a backpropagation neural network (BPNN) model for the early screening and staging of melanoma. They stated that the screening and staging accuracies of the models 83% and 96%, respectively. Based on Kruskal–Wallis (KW) statistical analysis, Ca and Na are identified as biomarkers for early screening and staging, whereas K and Mg play significant roles in the staging of melanoma. Melikechi et al. (2016) [172] developed models (LDA and RF) for differentiating blood plasma specimens of mice suffering from ovarian carcinoma. The accuracy achieved by the two models (LDA 70–75% and RF 79–81%) for the three age-specific groups, 8-, 12-, and 16-week-old mice, is similar and considered comparable across all age groups.

### 3.7. Miscellaneous Cancer Studies

**Figure 6:**
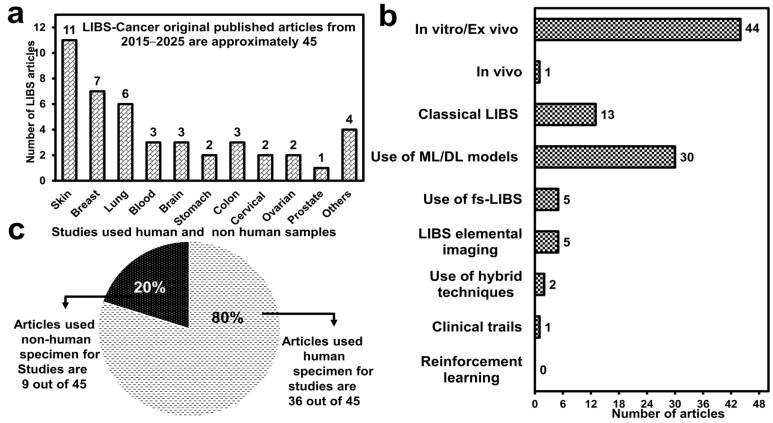
Distribution of journal-published articles on LIBS in cancer research for the period 2015–2025 (45 original articles). Statistical information about (**a**) number of articles related to different types of cancer; (**b**) adoption of methodologies and clinical trials; (**c**) number of studies performed on human and non-human specimens. Types of cancer and related references: skin [51,52,82,117,118,125,145,150,168,171,174], breast [35,47,127,162,163,169,175], lung [121,157,158,159,160,164], blood [48,119,166], brain [131,151,165], stomach [123,156], colon [50,81,120], cervical [31,161], ovarian [27,172], prostate [122], and others [39,83,132,153,199].

Yue et al. (2021) [27] evaluated the effect of data reduction methods (PCA and SelectKBest (SKB) on the performance of a LIBS-NN model for early-stage ovarian cancer screening. Spectral analysis was performed on 176 blood plasma samples from cancer patients, including ovarian cysts and normal cases. Essential electrolytes in blood plasma for preserving homeostasis in the body are metal elements (K, Na, Mg, and Ca), and an imbalance in their concentration indicates a state of abnormality in patients. Cancer detection sensitivity and specificity are expressed at up to 71% and 86%, respectively. Teng et al. (2020) [165] identified glioma (a brain tumour) from its surrounding tissues based on attributed biomarkers (Ca and Mg) to the progression of abnormality. Similarly, Mohammadimatin et al. (2023) [131] detect the same biomarkers for discriminating between two types of lethal brain cancers, glioblastoma multiforme (GBM) and oligodendroglioma (OG), and healthy infiltrated brain tissues by exploiting SD-LIBS. In addition to these types of cancers, several further studies related to oral, prostate, and cervical cancers are tabulated in Table 6 and Table 7. The respective experimental information of these studies can be found in Table 3. A statistical summary of the LIBS-cancer research is visualized in Figure 6 at the end of Section 3.

## 4. LIBS in Calcified Tissues Analysis

**Figure 7:**
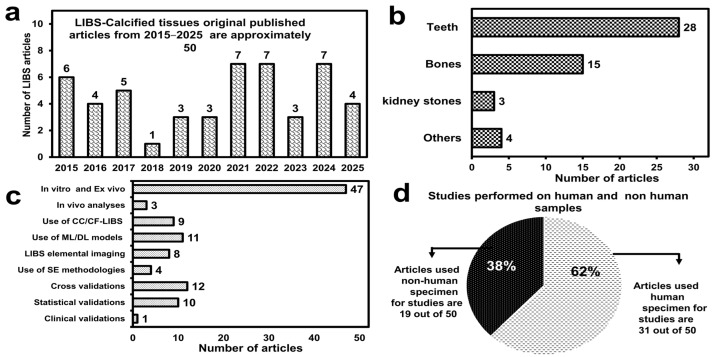
Summary of journal-published articles on LIBS of calcified tissues from 2015 to 2025 (50 original articles). Statistical information about (**a**) number of publications per year; (**b**) number of articles on teeth, bones, kidney stones, and others; (**c**) adoption of methodologies and validation procedures (cross, statistical, clinical); (**d**) number of studies performed on human and non-human tissues. Types of calcified tissue and related references: Teeth [80,84,106,107,112,124,130,147,201,202,203,204,205,206,207,210,211,212,213,216,217,219,221,229,230,231,232,237], bones [49,83,96,115,128,146,152,199,206,207,212,215,222,233,238], kidney stones [46,148,220], and others [234,235,236,239].

Calcified tissues are biological tissues that contain the mineral hydroxyapatite (HA, ${\mathrm{Ca}}_{10}{\left({\mathrm{PO}}_{4}\right)}_{6}{\left(\mathrm{OH}\right)}_{2}$). According to the definition, primarily teeth and bones are calcified. Their characteristics, including biocompatibility, slow degradation, structural support, and metabolic functions, allow them to participate in biological processes. Additionally, their chemical compositional understanding offers insight into physiology and pathological conditions [102,200]. LIBS has been exploited for such purpose; since the last decade, a variety of studies have been performed with improvisations such as the following: (i) LIBS has been employed to quantify the relative intensity of various elements, enabling detailed elemental profiling of biological and inorganic materials including studies on teeth [201,202,203,204,205,206,207,208,209,210,211,212,213], bones [79,97,214,215], and gallbladder stones [148]; (ii) investigators have integrated LIBS spectral profiles to compute area to determine the ablation thresholds of dental tissues [216,217,218], providing critical insights into laser–tissue interactions; (iii) LIBS is used to study laser-induced plasma parameters, for understanding the physicochemical properties of dental tissues [203,204,205,219] and bones [97]; (iv) CF-LIBS framework has been applied for the quantitative analysis of bones [49]; (v) CC-LIBS has been utilised to establish calibration curves for teeth [124,130] and kidney stones [220], enhancing the accuracy of elemental quantification; and (vi) LIBS signal enhancement methods NE-LIBS have been integrated to improve signal intensity for teeth [130] and bones [128], increasing the sensitivity and precision of measurements. It has also been employed for spatial elemental mapping of teeth [221], providing detailed distribution patterns of elements within the tissue (vii) LIBS data has been combined with ML algorithms to enhance the analysis of teeth [84,96,112,147], and bones [96,128,146,152], enabling automated classification, pattern recognition, and predictive modelling (viii) The use of ultrafast femtosecond lasers in LIBS has been explored to examine teeth [84] and bones [222], offering higher precision and reduced thermal damage compared to conventional nanosecond LIBS. Table 8 (in Section 4.1) and Table 9 (in Section 4.3) categorise and summarise the LIBS research work on human and animal calcified tissues, respectively. In addition, Figure 7 (at the end of Section 4.3) gives a statistical overview of recent LIBS research on calcified tissues.

### 4.1. Evolution of LIBS in Dentistry

In 1964, the first research on using lasers as a surgical tool in dentistry was published; at that time, the energetic pulsed ruby laser was used to eradicate caries [223]. These preliminary studies define fundamental concepts of laser dental interaction. However, its practical applications were not permitted due to the thermal damage induced in dental tissues by the available lasers in the early 1960s. Therefore, further research was carried out, leading to a significant breakthrough in this field of study with the development of laser technology in subsequent decades. Particularly, ultra-short laser pulses have proven to be practically valuable by enabling precise control over the ablation of carious tissues, allowing for the ablation of dental fillings without damaging the surrounding healthier tissues, and providing online feedback of endodontic treatment via laser-induced plasma-emitted radiations. The latter aspect is that when LIBS is introduced into dental studies to inspect the degree of thermal, mechanical, and optical damage induced in teeth by lasers. Successive LIBS research has been conducted to exploit plasma-emitted radiations as a diagnostic parameter for online monitoring of caries removal processes. Teeth are the most suitable sample for LIBS analysis, composed of four tissues: three of them, enamel, dentin, and cementum, are hard, while the fourth is soft, known as pulp. The outer layer, enamel, contains ~95 wt% hydroxyapatite (HA), ~4 wt% water, and only ~1 wt% organic substances; the middle layer, dentin, is comprised of ~70 wt% HA, ~10 wt% water and ~20 wt% organic matter; tooth root covering tissue cementum is least mineralised with ~45 wt% hydroxyapatite, ~35 wt% organic matter, and ~20 wt% water; the inner part, pulp, is non-mineralised and primarily composed of blood vessels and nerves [224]. The fundamental building block of teeth is HA crystal; its presence was confirmed by LIBS detection of its constituent elements, as evidenced by intense emission lines of Ca, P, O, and H [204,217].

Caries is the most common reason for dental infections (pericoronitis, periodontitis, pulpitis, osteomyelitis, gingivitis, cellulitis, and cracked tooth syndrome) caused by the quick reproduction of pathogens (bacteria and viruses) on tooth surfaces that lead to the demineralisation of dental tissues [16]. Caries alter the dental elemental composition, and loss of Ca is substituted by other cations detected by the LIBS analytical technique. There is no considerable relation between chemical properties and the type of teeth. However, it is one of the requirements to know the chemical profile of teeth elements to design a novel class of biomaterials for clinical purposes [225]. It is essential to understand the role of elements in human teeth to comprehend the purpose behind their detection. Multiple trace elements are indispensable for human growth, particularly in ensuring normal tooth development. Excess or deficiency of elements can be detrimental to the body, having a direct association with environmental conditions, lifestyle habits (such as diet and drug use), and diseases (diabetes, hypertension) [226]. These elements enter to the human body and are deposited into the tooth, causing demineralisation or decalcification processes that primarily lead to tooth decay due to a deficiency of calcium ions. Trace elements must be within the permissible limit provided by WHO; otherwise, adverse effects on teeth have been reported [227].

LIBS can discriminate between healthy and carious tissues by determining plasma parameters and elemental variations produced by caries-induced demineralisation, which are matched with the constituent concentrations of healthy tissues or standard references. Several such studies exist in the literature and are reported in this article: Khalid et al. (2015) [211] measured plasma parameters and elemental composition of healthy and carious tooth tissues (enamel, dentin, and cementum). They reported that Ca concentration is lower in carious enamel than in healthy ones compared with the artificially fabricated pellet reference (CaCO_3_). The highest contents of microminerals (Pb, Sr, Zn, and Fe) and low concentrations of Ca were found in enamel and turned into the most carious-affected tissues among other dental tissues. Batool et al. (2021) [204] recently measured the temperature and electron density of an Nd:YAG laser with both its fundamental and second harmonic outputs. They concluded that Nd:YAG laser in the second harmonic mode induced less temperature on the enamel surface and higher electron density than its fundamental mode. In laser dentistry, the second-harmonic Nd:YAG laser is declared as a suitable candidate for cavity preparation, as it produces well-defined ablations while preserving adjacent healthy tissue. The observed effects correlate with distinct electron densities between laser modes, corresponding to their emitted photon energies.

The study by Gazmeh et al. (2015) [36] demonstrated that LIBS combined with PLS-DA could effectively distinguish between sound teeth and those with caries. It was worthwhile to use statistical analysis to classify samples because there was little difference in spectral line intensities (P, Ca, Mg, Z, Sr, C, Na, H, and O) between healthy and carious tissues. Elemental emission line intensities are considered variables to build the model. The author claims high model prediction accuracy in classifying unknown samples (healthy and carious tooth samples. Hadeethi et al. (2016) [216] and Mustafa et al. (2022) [217,218] have measured the dependence of Ca emission intensities in dental tissues (enamel, dentin) on the fluence of the laser pulse using an Nd:YAG laser-based LIBS. The thresholds of laser fluence for calcium in enamel and dentin tissue were found to be 1.41 J/cm^2^ and 0.38 J/cm^2^, respectively. In the case of enamel, a higher threshold laser fluence is required for Ca emission in enamel than in dentine due to its calcified nature and rigid structure. To date, the literature lacks sufficient data on the structural and chemical composition changes in teeth associated with ageing. It cannot be advised to children and adults on similar preventive measures and treatment procedures in modern dentistry. Therefore, age-specific morphological and chemical analyses of teeth are strongly recommended to support the development of optimised materials, including anti-caries hygiene products and dental filling materials [228]. Briefly, 28 LIBS-human dental research works, along with 5 studies on human bones and kidney stone examination, are summarised chronologically in Table 8.

**Table 8:** LIBS analyses of human calcified tissues.

Tissues	Ref.	Purpose/Application	Method	Findings	Comments
Teeth	[221]	Imaging of teeth using long-pulse laser to evaluate LIBS performance/dental anatomy	LIBS elemental imaging	Visualise variation in elemental distribution from enamel to dentine	Limited to major elements, alternative needs to be explored for trace elements
	[106]	Migration of species from dental restorative materials to tooth matrix/clinical applications (pathological identifications)	Measuring line intensities	One source of trace elemental accumulation in tooth matrix is dental filling materials.	Hg in healthy tissues not detected, LOD higher than concentration (0.10 μg g^−1^)
	[124]	Elemental quantification of teeth using appropriate calibration method/pre-clinical dentistry	CC-LIBS	Quantification of Ca, Mg, C, and Zn, error rate measured for different methods	Considerable error range for LIBS with respect to standard techniques (ICP-MS, EDX); inaccuracies due to matrix effect
	[130]	Effect of NPs deposited on teeth on LIBS performance/nano-dentistry	CC-LIBS	Signal enhancement and improved calibration for Ca with ZnO NP	Control of size, shape, and distribution of NPs difficult; reproducibility is of concern
	[147]	Sex identification/odontology	LIBS-ANN	Ca, Mg, P, Sr, and H used for classification; accuracy for males 98%, for females 99%	Small sample size (n = 10) and limited number of spectra (N = 500) are insufficient for definitive conclusion
	[201]	Classification of teeth according to age and sex for healthy and carious teeth/pre-clinical dentistry	Intensity ratio of spectral lines	High concentration of Mg and Pb in carious teeth; trace elements: intensity decreases with age, female teeth have higher intensity	Small sample size (n = 8); no statistical, cross, and clinical validation performed
	[205]	Effect of laser wavelength and irradiance on plasma parameters/pre-clinical dentistry	Boltzmann and Saha–Boltzmann plot methods	High plasma temperature at 1064 nm; plasma frequency and Debye length increase with irradiance	Limited clinical relevance; results published earlier confirmed
	[219]	Monitoring the migration of dental filling material into tooth/laser dentistry	Intensity ratio and Stark broadening methods	Plasma temperatures for composite, dentine, and amalgam are different	Element concentration not quantified; limited to ex vivo analysis; other relevant materials are to be analysed
	[213]	Effect of antimicrobial agents on remineralisation of dentine/tissue restoration	Changes in intensity vs. wavelength emission profiles	Biomimetic remineralisation of carious dentine by activating an antimicrobial agent using fs laser	Use of different materials will change results (matrix effect); no statistical cross validation and clinical validation
	[229]	Variation in elemental composition in ankylotic tissues/orthodontic	Elemental imaging	Ankylotic tissues have higher concentration of Ca and P	Limited spatial resolution of 30 µm (Ca, Mg, P); role of Mg is to be explored
	[217]	Variation of Ca and Mg in dental tissues after laser irradiation/laser dentistry	Integrated peak area vs. laser energy density	Threshold laser fluence for Ca in enamel and dentin determined	Threshold laser fluence for Mg not detected (instrumental limitations)
	[230]	Age and sex identification/orthodontic treatments	Measured average intensities	Ca, P, and Fe concentrations decrease with age, higher concentrations in females than males	Variation is linked to orthodontic abnormalities; cross validation and clinical validation should be considered
	[112]	Diagnosis of dental pathologies/pathological identification	Relative intensity	Ca/P ratio decreases in presence of plaque	Ca/P signal range is close for healthy and pathological regions
	[204]	Characterisation of dental tissues/clinical dentistry (dental implants and cavity preparation)	intensity ratio and Stark broadening methods	Plasma temperature higher and electron density lower for Nd:YAG laser wavelength 1064 nm than for 532 nm	Outcomes are to validated by practitioners using other lasers (CO_2_, diode, Er:YAG) used in laser dentistry
	[107]	Assess interaction of laser beam with dental tissues and dental material/dental health and safety	LIBS and photoacoustic sensor	More intense plasma on carious than on healthy tissues. Release of toxic elements (Hg, Ag, Cu, Sn) from amalgam hazardous	Used approximation for acoustic wave propagation may not be appropriate for clinical applications
	[202]	Detect early signs of osteoporosis in periodontal patients/clinical dentistry	Mean spectral intensity	Lower (Ca) and higher (K, Mg) content associated with osteoporotic group compared to control group	Control group had periodontal disease, which can have specific effects of osteoporosis (on periodontal tissues)
	[231]	Examination of dental tissues, enamel (apical and buccal) and dentine/pre-clinical dentistry	Intensity ratio of spectral lines	Apical enamel is the hardest among buccal enamel and dentine	No statistical, cross, and clinical validation; in vitro analysis
	[84]	Thermal effect of fs laser ablation on teeth for caries removal/laser surgery	Simulation method (thermal model)	Minimal thermal damage to surrounding nerve tissues; acceptable removal rates	Optimisation of laser fluence below carbonization threshold of each tissue is challenging
	[80]	Evaluation of diffusion of mercury to dental tissues/tooth restoration	Fs-LIBS	Hg penetration depth for deciduous and permanent teeth determined	Spatial resolution limited to 100 µm; penetration depths of other metals in tissues are to be determined
	[216]	Ablation threshold fluence for enamel and dentine/laser dentistry	Emission intensity vs. laser energy density	Ablation threshold for enamel and dentine determined	Ablation damages tissues inducing variations in surface topography and structural morphology
	[232]	Elemental variations in teeth associated with cariogenic and periodontal pathologies/oral surgery	LIBS intensity of emission lines	Higher concentration of C, O, K, F, Na aggregates in periodontal teeth compared to those with cavities	Statistical cross validation and clinical validations to be done; in vitro analysis
	[203]	Detection of toxic elements in smokers, non-smokers, and teeth/periodontal probing	Calibration curve for quantification	Higher concentration of hazardous elements (Pb, Cd, As) in smoker group than in non-smoker group.	No statistical analysis performed; correlation of results to smoking habits or chronic periodontitis difficult
	[211]	Examination of deciduous teeth to measure toxic elements/dental health and safety	Comparing spectra with matrix-matched references	High amounts of toxic elements in enamel make it the most affected tissue in the tooth	Small sample size (n = 4); no statistical, cross, and clinical validation; in vitro analysis
	[210]	Improve sensitivity of system for caries identification/optical feedback in laser dentistry	Measuring intensity variations	Intensity ratio Zn/Ca increases in presence of caries	Use of two different lasers (Nd:YAG, Er:YAG) makes treatment difficult
Bone	[152]	Performance evaluation of ML models in bone classification/pre-orthopaedic surgery	LIBS-ML (PLS-DA, LDA, LR, SVM, SIMCA, CART, NN)	NN has exceptional performance in terms of sensitivity, robustness, generalisation	Accuracy of NN (100%) may be due to overfitting; other models’ accuracy is 42–66%
	[83]	Discriminate between normal and pathological bone/optical feedback in orthopaedic surgery	Fs-LIBS-PCA	Higher Mg intensity relative to Ca in pathological bone compared to normal bone	Unable to detect bones suffering from severe pathologies
Kidney stones	[148]	Discrimination of gallbladder stones (mixed vs. pigment GB 2)/diagnosis	LIBS-PCA and PAS	Variation in intensity of Ca, Sr, K, and CN; absence of calcium phosphate in GB 2	Different sample preparation methods for LIBS and PAS measurements
	[46,220]	Measure major and trace elements in gallstones/diagnosis	LIBS/WD-XRF/FTIR	Trace element (Zn, Pb, Cr, Cd) concentration exceeded safety limit	LIBS LOD for trace element 10–19 ppm

### 4.2. LIBS in Bone and Kidney Stones Analysis

The major chemical compounds in bones are ~69 wt% hydroxyapatite, ~10 wt% water, ~20 wt% collagen, and ~1 wt% proteins [224]. Physiological bone assessment is of great importance in defining therapeutic procedures. To detect irregularities, it is necessary to measure imbalances in chemical compositions and observe morphological alterations in cases of bone abnormalities. Moncayo et al. [152] evaluated LIBS-ML algorithms (soft independent modelling of class analogy (SIMCA), PLS-DA, LDA, classification and regression trees (CART), LR, SVM, and NN) for their classification accuracy on human bone samples. NN performance is relatively satisfactory in discriminating highly similar human bones. Ruby et al. [83] proposed fs-LIBS as a feedback control system for the removal of primary bone tumour, where a higher Mg peak intensity relative to Ca is related to abnormality. But the study is unable to define diagnostic criteria for secondary bone tumour due to their inherent heterogeneity. Overlapping spectra on the PCA plot indicate that fs-LIBS may not be appropriate for late-stage tumour detection. Alternatives need to be explored by refining sample preparation strategy and spatial mapping of LIBS signals. Several articles on LIBS-bone analysis are available in the literature for archaeological, environmental, and forensic applications; however, from a health and medical perspective, their application to human bone specimens is rare.

Zainab et al. [148] performed compositional analysis on two gallbladder stones (mixed (GB 1) and pigment (GB 2)) using two spectroscopic techniques: LIBS for elemental analysis and Photoacoustic spectroscopy (PAS) for molecular information. LIBS-PCA was performed based on the variation in emission intensity of classifiers Ca, Sr, K, and CN for GB 1 and GB 2 to classify them. PAS molecular analysis of GB 1 (calcium carbonate, calcium phosphate, bile acid, bilirubin, and fatty acid) and GB 2 (calcium carbonate, bile acid, bilirubin, and fatty acid) revealed the absence of calcium phosphate in the latter. Gondal et al. [220] quantified carcinogenic metal content in three kidney stones (extracted from three different patients) using SP-LIBS with Nd:YAG laser (266 nm, 8 ns, 20 Hz, 25–50 mJ). Calibration curves are drawn to quantify the concentration of detected metals (Ca, Zn, Cr, Cd, and Pb), and LIBS outcomes are validated with ICP-MS. They concluded that metal concentrations in stones are beyond the safety limits of U.S. Environmental Protection Agency (EPA) and Food and Drug Administration (FDA). Pilot studies [148,220] have yet to demonstrate clinical implications, so they require further extension to broader populations, including diverse demographics, various stone compositions, and different ethnic groups.

### 4.3. LIBS in Animal Calcified Tissues

Beverage consumption is trending for humanity’s pleasure, relief, and to maintain energetic health. Its effect on teeth was enlightened by Manno et al. (2018, 2020) [206,207]. The impact of coffee immersion on rat teeth (in vitro) was investigated through a comparative study of coffee consumption by different age groups and their respective age-matched control groups. Coffee can induce decalcification of teeth, leading to erosion and exposing the dentin by thinning the enamel layer. In vivo and in vitro, hot and cold beverages (green tea and water) influenced rat teeth. Drinking green tea at room temperature protects teeth against erosion. However, caution is required when consuming hot tea, as it can cause enamel degradation due to the interaction between hot green tea catechins and hydroxyapatite (HA) in the teeth. To reinforce this, atomic force microscopy (AFM) is combined with scanning electron microscopy (SEM) to evaluate surface topographic characteristics and quantify erosion. Refraining from hot beverages reduces direct exposure to teeth, consequently preserving the dental structure, making them less likely to decay, and ensuring their long life [206].

Tariq et al. [233] evaluate the hardness of calcium-rich tissue by measuring the Ca/P ratio of HA (extracted from bovine bone) using laser-induced plasma emission spectral lines. They reported a relative error of <6% between LIBS and EDX findings for Ca/P. Although high accuracy and repeatability in measurements were obtained and verified by varying plasma, further research studies are required to verify the authenticity of using LIBS to determine Ca/P.

Roldan et al. [49] applied CF-LIBS for elemental analysis of rib bone (wild boar) by measuring plasma temperature and electron number density. Spectral analyses were performed across the ultraviolet to near-infrared (UV-NIR) and vacuum ultraviolet (VUV) ranges, as light elements are challenging to detect in UV-NIR due to the low signal-to-noise ratio for emission lines of such elements within a complex matrix. A broad spectral range enabled the detection of more elemental emission lines; even P is detected in the VUV region, further allowing the calculation of the atomic ratio of Ca/P (1.6 ± 0.4) for a gate delay of 500 ns to assess bone hardness. Salam et al. [128] utilised NE-LIBS, biosynthesised Ag-NPs sprinkled on animal feed and bovine bone (ancient and modern) samples to enhance the emission intensity of spectral lines in NE-LIBS spectra. PCA was applied to the data to distinguish between bone and fodder types for assessing the feeding strategy of livestock. Spectrochemical analytical data revealed the presence of numerous common elements in bones and feed that can inform feeding strategies for animals regarding their health throughout their lifetime. Additionally, this study can be beneficial for human health, as it depends on farm animals as their primary food source.

**Table 9:** LIBS analysis of animal calcified tissues.

Tissues	Ref.	Purpose/ Application	Method	Findings	Comments
Rat dental tissues	[206,207]	Effect of beverages on rat dental tissues/veterinary dental care	Line intensity ratio	Coffee induces loss of Ca and P; decalcification of enamel	Molecular mechanism of enamel protection by green tea needs to be assessed
Chameleon oral tissues (tooth and bone)	[212]	Age-related variation in tooth-bone fusion area/physiological condition	Elemental imaging	Intensity of Ca, P, and Mg correlated with age in junction area of tooth and bone	Comparative analysis required to support claim that ankylosis is pathological state in mammals but is physiological for chameleon
Pig bones	[215]	Effect of acidic environment on bones/physiological state	Line intensity ratio	Intensity of Ca II/Ca I increases with concentration of sulfuric acid and time; hardness of bone surface is associated to calcium sulphate	Clinical relevance not demonstrated
Porcine bones (femora)	[115,146]	Differentiate hard (bone) and soft tissues (muscle and fat)/laser osteotomy	FO-LIBS-ML	Sensitivity of canonical DFA model for differentiating fat (84.6%) is lower than that of bone (100%)	Significant difference in model’s accuracy for soft tissue analysis revealed limitations of LIBS for such tissue examination
Boar bones (rib)	[49]	Analysis of light elements/Orthopaedic	CF-LIBS	Ratio of Ca/P measured	Difficult to fulfil prerequisites for CF-LIBS of biological samples; uncertainties of plasma parameters; comparative validation required
Bovine bones (femur)	[128]	Discrimination of different bones and different fodders/health and medical	NE-LIBS-PCA	Deposition of Ag NPs on bones and fodder enhances sensitivity of LIBS	Aging of bone and metabolism of cattle affect element concentration; complicates correlation of feeding strategy and bone composition
Pig (oral mucosa, peripheral nerve, dental pulp, dentine, enamel, cortical bone, cancellous bone)	[96]	Differentiation of hard and soft tissues/laser surgery	LIBS-PCA-LDA	Ratio of Ca/C identified as classifier (higher in hard tissues than in soft ones)	Sensitivity and specificity of LDA below 70% for enamel-dentine pair classification is serious issue in hard tissues examination (matrix effect lower than in soft tissues)
Bovine (bones and muscles)	[222]	Effect of laser repetition rate on ablation of dry bone/laser surgery	Fs-LIBS	Higher repetition rate allows fast cutting of bone (fluence above thresholds)	Temperature rise during ablation of bone should not exceed threshold for irreversible damage of nerves
Rat bones (thigh)	[97]	Role of lead in bone/animal health and medical science	Relative line intensity	Ca and Mg signals decrease with increasing Pb concentration	Error (18%) of measured plasma parameters high; quantification of elements hindered
Chicken eggshells	[234]	Discrimination of organic eggs from inorganic/food quality control: health and safety	LIBS-NN	Classification accuracy of NN for eggshell across various groups is 100%	Research focused on Ca; quantitative analysis of other elements (Mg, Fe, Al, Sr, Zn, Mn) to be performed
Chicken eggshells	[235]	Measurement of plasma parameters/pre-veterinary	Boltzmann plot and Stark broadening	Determination of temperature and electron number density and of inverse Bremsstrahlung absorption coefficient	Large uncertainty (5–50%) of used transition probability data limits precision of determined parameters; practical application is lacking
Chicken eggshells	[236]	Determination of heavy and trace elements in organic and regular chicken shell/food quality control: health and safety	CF-LIBS (OLCF and SC)	Ba detected only in organic shell; SC-LIBS is more reliable for quantification of heavy metals and trace elements	LOD values for toxic metals (Pb, Cd, Hg, As) remain uncertain

Abbasi et al. [115] explored LIBS as a feedback system for differentiating bone from soft tissues during laser-osteotomy, aiming to improve surgical precision and safety. The classification of three sample groups (bone, muscle, and fat) based on the intensity ratio of selected peaks was performed using discriminant functional analysis (DFA). The ROC curve analysis depicted an accuracy of 99% for hard–soft tissues (bone–muscle and bone–fat) and a relatively lower accuracy (90%) associated with soft tissues (muscle–fat). When ex vivo results are applied to in vivo analysis, the presence of various biological fluids and dynamic physiological conditions in a living body presents significant challenges that are not addressed in this study.

Ying et al. [239] used LIBS to track the shell growth in sea shells (ezo scallop shell) from biological perspectives. They proposed that Ca intensity is constant on the shell surface and can be used as an internal reference, whereas Sr content increases with the growth of the shell. Applications of LIBS in different animal tissues (teeth, bones, and eggshells) from medical perspectives are tabulated in Table 9.

## 5. LIBS Hybrid Technology

**Figure 8:**
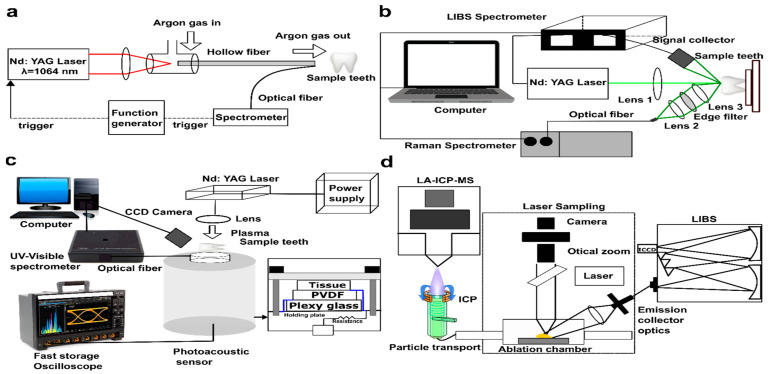
Schematic design of LIBS coupled with (**a**) optical fibre (adapted from [210] with permission); (**b**) RS (adapted from [42] with permission); (**c**) polyvinylidene fluoride (PVDF) based photoacoustic sensor (adapted from [107] with permission); (**d**) LA-ICP-MS (adapted from [240] with permission).

Biomedical examination requires a comprehensive analysis of specimens, encompassing their elemental, molecular, and structural components. However, it is challenging to obtain such detailed information using standalone techniques due to their limitations. Hence, hybrid models in which two or three methods are integrated into a single device are extensively used to obtain comprehensive information and achieve the best possible outcomes [42]. LIBS can be established as a diagnostic tool that complements other analysis techniques, such as RS and ICP-MS, rather than replacing them [68]. For a better understanding, some structural designs of LIBS-coupled techniques used in various medical applications are illustrated in Figure 8.

Integration of LIBS and Raman technology enables elemental and molecular information about tissue composition. Khan et al. (2022) [174] fused LIBS and Raman spectral data for the classification of melanoma tissues using ML models (ELM, PLS-DA, KNN). The higher intensity of Mg in the LIBS spectra and the spectral shift related to amide III and lipid molecules in the Raman spectra of tumour tissues are identified as biomarkers. Matrix effect should be addressed when tissues were collected from different body parts (lymph node and skin). It highlights the issue of confounding results, as spectral differences can reflect the anatomical site rather than the state of cancer alone. The reported average classification accuracy for ELM is 99.3%, which raises concerns about overfitting due to the small data set (10 samples from 2 patients). Additionally, statistical cross-validation and clinical validation are required for more reliable analysis. Lin et al. (2024) [121] used a bimodal approach (LIBS + Raman) to improve the diagnostic accuracy of the CNN model. For this purpose, LIBS (Ca, Mg, Fe, and Cu) and Raman (phenylalanine, tyrosine, tryptophan, amide III, and protein) identifiers were measured for lung cancer staging. These biomarkers are not cancer-specific and can vary due to obvious reasons (dietary habits, environmental conditions, and tissue heterogeneity), which may potentially confound staging accuracy. The bimodal information is combined using a decision-level Bayesian model, which enhances the classification efficacy up to 99% in the CNN framework. The model may oversimplify the biological interaction, and the results need to be validated against the gold standards (histopathology, immunochemistry, and CT).

Batch effect is caused by operator variability and instrumental variations, consequently reducing the performance of diagnostic models. Shi et al. (2025) [163] fused LIBS and FTIR spectral data from serum samples of breast cancer patients to enhance the detection ability of CNN and GRAN by correcting the batch effect. Classifiers from LIBS (Na, Ca, and Mg) and FTIR (Amide 1, II, and A) were identified. Although technical details of the algorithms are not provided, an in-depth knowledge of data science is required to understand the mechanism behind the improvement. GRAN model accuracy in detection is 89% and validating the model in a clinical setting at a large scale is recommended for generalisation.

Sasazawa et al. (2015) [210] developed an optical-fibre-based LIBS system combined with coaxial Argon gas flow aiming for in vivo analysis of carious enamel, as illustrated in Figure 8a. Specimens are segregated into three groups based on the level of decay: (i) early stages of decay (decay and cavities stayed only on enamel), (ii) advanced stages of decay (cavities reached dentin), and (iii) healthy teeth (without decay or cavities). Two different lasers are used for experimentation: conventional dental Er:YAG laser (2.94 µm) is used for ablation purposes, and Nd:YAG laser (1064 nm) for LIBS analysis. Zn is strongly detected in an early stage of dental decay and considered a biomarker of decayed teeth. The intensity ratio of Zn/Ca increases as caries develops, reaching a high of 0.013 for dentin caries, whereas lowest is 0.0044 for healthy teeth. However, there is doubt about the presence of Zn whether it originates from stains or dietary habits, and the method is unable to detect it in dentine caries. Research lacks histological confirmations, and reliance on a single element for treatment is quite risky. These issues need to be addressed before transitioning from in vitro to in vivo analyses.

Khosroshahi et al. (2020) [107] designed LIBS-integrated photoacoustic (PA) sensors to measure the safety of laser-dental treatments, as shown in Figure 8c. Polyvinylidene fluoride (PVDF) is a common material used in biomedical applications as a PA sensor. It is used to detect the corresponding stress waves generated by heating as a result of photon absorption and propagating through the material. LIBS-PA provides insights into the mechanical effects and the elemental compositional changes that occur during laser interaction. Pressure due to thermal waves increases non-linearly with laser fluence, and a maximum pressure of 8 kPa was measured for healthy teeth. Plasma colour varies with pulse numbers, indicating different temperatures of ejecta. In relation to healthy teeth, the formation of intense plasma in carious teeth is attributed to the presence of C and Sr. In the case of amalgam combustion, the intense emissions of Hg, Ag, Cu, and Sn lead to a high plasma temperature of 15,000 K. Inhaling these heavy metal elements causes serious health problems. The limitations of this research include visual sample inspection, a large variation in plasma temperature (590–15,000 K) without measurement of errors, ex vivo analyses, several assumptions, and a lack of clinical relevance, which may prevent this system from being adopted in dental clinics.

## 6. Limitations, Possible Solutions, and Recommendations

Spectrochemical analysis, including LIBS, faces several challenges, such as background drift, low signal-to-noise ratio, peak shifting, peak broadening or narrowing, and peak overlapping, which affect the sensitivity, specificity, stability, and reproducibility of analytical signals [142]. Potential causes of these issues include non-optimised laser parameters, variation in ablation efficiency, non-ideal plasma conditions, the transient nature of plasma, matrix effects, fractionation, spectral absorption, heterogeneous sample, complex data analysis, instrumental limitations, and environmental instrumental influences. It is necessary to resolve the above issues to enhance the diagnostic properties of LIBS and fully realise its potential as a stand-alone or complementary technique. Many thousands of laser shots can be beneficial in addressing sample inhomogeneity issues. Pre-sample treatment has improved LIBS signal sensitivity by intensifying plasma emission, particularly in biological studies [241]. In addition, several potential approaches (mentioned in Section 2.1) can be used to minimise matrix effects.

AI-assisted LIBS methodology in diagnosis requires expertise to handle complex data and avoid poor feature selection, which can lead to overfitting. Models’ generalisability is missing due to the choice of a smaller sample size. They often lack interpretability, which makes it challenging to correlate spectral features with biological specimens [52]. Despite encouraging outcomes of ML models being reported, many studies offer limited discussion on model interpretability and potential clinical integration. Thus, future research should prioritise transparent model evaluation, cross-institutional validation, and alignment with clinical diagnostic requirements to ensure robustness and translational value. Reinforcement learning (RL) and imitation learning (IL) algorithms can explain causal effects, provided that the system designs in this context are suitable. The approach has the potential for more accurate disease classification, improved efficiency of treatment procedures, and personalised medical prescriptions [181].

Descriptions of multiple studies suggest that investigators still lack a unified understanding of which abnormalities exhibit variations in element composition or the extent to which these variations occur. It has been more than two decades since LIBS detected the first cancer, and it is still in a research phase that requires confirmations, establishing protocols, and consulting with experts. Most studies are conducted in vitro, which means specimens are extracted from the body, and characterisation is performed in a laboratory under controlled conditions. The issue is whether their findings can be applied to in vivo analysis in real-world scenarios. The studies are recommended where the researchers reproduce their results fairly on real patients. The incorporation of improved spectroscopic instruments, LIBS configurations, multiple LIBS hyphenated techniques, and LIBS-ML has tremendous potential to overcome the shortcomings of LIBS for diagnostic applications. The handheld LIBS technology needs to be upgraded to eliminate the limitations that prevent it from providing the desired features for biological sample diagnosis, including in-depth profiling, in situ detection capability, a micro-destructive nature, minimal or no sample preparation, and the ability to perform in vivo analysis under ambient conditions [242].

Despite advancements in cancer research, certain cancers, including pancreatic, paediatric, gynaecologic, prostrate, neuroendocrine, calcified tissue cancer, rare cancers, penile, anal, salivary gland, and small intestine, have not been extensively studied via LIBS. Similarly, a range of calcified tissues, including calcified tendons and ligaments, dermal bone structures, coral skeletons, exoskeletons, eggshells, and otoliths, can be analysed using LIBS technology to fill existing research gaps and understand the compositional heterogeneity of these tissues. Ideally and potentially, LIBS in vivo surgery is possible for surgeons and dentists, allowing them to ablate tumours or carious tissues while analysing ablation events and categorising spectroscopically, shot by shot, to obtain real-time feedback on the tissue being removed.

## 7. Conclusions

LIBS is an innovative chemical elemental analysis technique progressing in diverse research fields, including biomedical sciences. It is a well-established technique from a qualitative perspective, but reaching quantitative goals for biological specimens requires considerable effort to overcome the current challenges of self-absorption and matrix effects. Identifying elemental biomarkers associated with cancer pathologies and calcified tissues helps in early disease diagnosis and adaptive therapeutic procedures, ultimately reducing mortality rates. Standalone LIBS cannot achieve exemplary results because pathological identifiers are in minute quantities. Therefore, employing signal-enhanced variants (e.g., DP-LIBS, EF-LIBS, and NE-LIBS) for spectral collection, advanced data processing models for spectral analysis, and ultrashort fs LIBS for high resolution elemental imaging are significant. In DL architectures, integration of reinforcement learning and transfer learning methods can provide more realistic data interpretation. LIBS, in conjunction with medical equipment, can provide advanced diagnostic capabilities for diseases. An analysis of published data reveals that the application of LIBS in cancer research and studies on calcified tissues remains limited, with approximately 45 and 50 original articles published in high-ranking journals, respectively, between 2015 and 2025. Small sample size (n) in cancer studies ranging from n = 2 to n = 35 [31,39,50,81,117,119,120,123,125,131,150,153,164,165,166] and calcified tissues investigation ranging from n = 1 to n = 15 [49,96,97,124,128,130,146,147,201,206,211,220,222] is insufficient for robust statistical analysis to draw a definitive conclusion. To date, no studies have been identified that simultaneously validate LIBS outcomes across all three statistical, medical, and clinical domains. Most research articles lack medical validation of LIBS results (comparison of outcomes with standard diagnostics methods MRI, biopsy, and histopathology) and clinical validation (usage of tools in hospitals on real patient populations). Extensive in vivo research is required for cancer diagnosis and calcified tissue analysis, utilising large samples and incorporating appropriate theoretical modelling, statistical analysis with causal inference, and medical relevance to establish standardisation protocols and guidelines. LIBS cannot be applied in real biomedical applications, particularly in hospitals, until it proposes more reliable analysis for complex biological tissues compared to many mature elemental techniques that are available and considered standard reference techniques (AAS, LA-ICP-MS, and XRF). LIBS-hyphenated techniques can be combined with optical spectroscopic techniques and medical equipment to achieve diagnostic efficiency. It is available in physicochemical laboratories, and analytical results are reliable. There is perseverance in transferring LIBS from lab-based research to real medical diagnostic applications. Ideally, LIBS is envisioned to be in the hands of doctors, nurses, pharmacists, and practitioners within the next couple of years or more; however, in reality, challenges can persist in the field and pose significant obstacles to unleashing its true potential.

## Figures and Tables

**Table 1:** Influence of matrix effect (ME), challenges to LIBS analyses of human and animal tissues (hard and soft).

	Material/Tissues	Matrix Elements/Non-Matrix Elements	Challenges	Comments	Ref.
**Human samples**	Skin tissues (cancerous and healthy)	C, N, H, O/Ca, Mg, Na, K	Limited lateral resolution, unable to map trace elements with precision	Ratio of intensities used for standardisation to eliminate ME; spatial resolution in LIBS imaging lower than for LA-ICP-MS	[51,117]
	Skin tissues	C, N, H, O/Ca, Mg, K, P, Fe, Na	Spectral fluctuation	Extensive pre-processing methods (standard normal variate, autoscaling, auto centring, normalisation by area) used to enhance model efficiencies	[118]
	Blood	C, N, O, H, Ca, P/Fe, K, Na, Mg	Spectral fluctuation	ME of filter paper substrate and blood add up; signals of filter paper not subtracted, normalisation of intensities insufficient	[119]
	Serum	K, Na, Ca, Mg/Zn, Cu	Poor signal-to-noise ratio (SNR)	Self-absorption factor (0.6) to be reduced for reliable analysis	[120]
	Lung tumour	C, N, H, O, P, S/Ca, Mg, Cu, Fe	Detection of anomalous spectra	Pre-processing and z-score method used to reduce fluctuation; uncertainty about variation in intensity (ME or abnormalities)	[121]
	Prostate malignant tissue	C, N, H, O/Na, Fe	Low intensities, high background noise	Trace elemental detection and quantification are lacking	[122]
	Gastric tissues	C, N, H, O, Ca, Na/Mg	Poor SNR	Biopsies and extensive sample preparation required	[123]
	Teeth	Ca, P/-	Poor quantification precision	Certified reference materials for dental tissues not available; plasma properties of tooth samples and reference materials considerably different	[124]
	Teeth	Ca, P/Al, Ba, Hg, Pb, Sr	High limit of detection for trace elements	Non-matrix elements from filling materials migrate to matrix, unreliable quantification	[106]
	Teeth, bones	Ca, P/Mg, Sr	Poor repeatability, LIBS-ML model inefficient for classification	LIBS-NN model cannot be generalised; different models used for classification, even for specimens having similar matrices	[116]
**Animal samples**	Soft pork tissues (fat, skin, and muscle)	C, N, H, O, K, Ca, Na/-	Spectral fluctuation	LIBS-SVM classification sensitivity (74%) for similar muscle tissues is limited	[114]
	Porcine organic tissues (liver, brain, kidney, heart, lung, and skeletal muscle)	C, N, H, O/Ca, Mg, Na, K, Fe	Poor quantitative analysis	Matrix-matched reference materials required; larger data set of animal tissues required for validation	[109]
	Porcine tissues (bone, muscle, and fat)	C, N, H, O/Ca, K, Na, Zn, Fe, Cl	Background noise	LIBS-DFA model sensitivity and specificity for discrimination of soft tissues (muscle and fat) 10% less than that for bones	[115]
	Pig’s soft tissues (fats and nerves)	C, N, H, O, P, S/Ca, Na, K	Poor classification accuracy	Model performances to be improved; ex vivo analysis may not be applicable for in vivo studies	[113]
	Rib bones of boar	Ca, P/Mg, Sr	Uncertainty of measured plasma parameters restricts accurate elemental quantification	Optimisation of experimental parameters (gate delay, gas pressure, spectral range); enhanced CF-LIBS performance for all matrix elements of all tissues uncertain	[49]
	Mice skin tissues (melanoma lesion and normal)	C, N, H, O, P/Mg	Low average spatial resolution due to instrumental limitations	Unable to discriminate between highly similar tissues; conventional histological imaging used for cross-validation	[125]

**Table 2:** Reported pitfalls of AI models for the interpretation of LIBS data in various cancer investigations, along with possible solutions.

Type of Cancer/Methods/Refs.	Pitfalls	Remarks
Gastric/LIBS-KNN/[156]	KNN: poor scalability, curse of dimensionality, lack of interpretability	Generalised additive models (GAM) and XGBoost could be better alternatives
Lungs/LIBS-PCA/[159]	PCA: compromises important features having low variations, ineffective for non-linear relationships	For non-linear issues, kernel PCA can be employed
Lungs/LIBS-1D-ResNet/[164]	1D ResNet: domain-sensitive means model trained on one type of spectrometer; for data from another spectrometer transfer learning methods required	Fine-tuning can be effective in resolving domain sensitivity; regularising techniques and weight loss functions can reduce over-fitting
Lungs/LIBS-KPCA/[157]	Kernel has its own critical hyperparameters for tuning bandwidth and their values are not reported; KPCA is sensitive to outliers	It can be useful for a non-linear data set when the goal is to get predictive outcomes without understanding the features
Lungs/LIBS-Bagged tree model/[160]	Bagged tree model suffers from overfitting when a smaller number of spectra (280) is used	Overfitting can be reduced by eliminating noise from the LIBS spectrum
Cervical/LIBS-SVM/[31]	SVM is kernel-dependent, wrong selection leads to poor accuracy; effective for binary classification, unable to deal with multiple stages of cancer	Use of cross-validation is recommended for kernel selection because they have different regularisation parameters
Cervical/LIBS-CNN/[161]	CNN decision interpretation is not convincing; vulnerable to adversarial attacks; difficult to handle and has poor robustness	Adversarial training and multiple CNNs can provide generalisation
Brain/LIBS-SNN/[151]	Heuristic algorithms (cuckoo search algorithm used) computationally challenging; new method, not yet applied in other LIBS-medical studies	Usage of neuromorphic hardware can increase computational speed; hybrid architectures (ANN-SNN or CNN-SNN) can be more effective
Brain/LIBS-SVM/[165]	Identification accuracy of SVM is low (~60%); least reliable in intraoperative tumour classifications	Bayesian optimisation method can be applied for tuning penalty and kernel function parameters instead of using particle swarm optimisation (PSO)
Ovarian/LIBS-BPNN/[27]	BPNN model detail activation functions, regularisation and training strategy (epochs, batch size, and learning rate) is missing	Model description is required for reproducing the results in other labs
Breast/LIBS-PSCC/[162]	Significant drop in performance of CNN when shared pre-processing module is removed (accuracy decreased from 90% to 82%); PSCNN outcomes are instrument-dependent	In limited scenarios, shared pre-processing can be effective
Blood/LIBS-RSM-LDA/[166]	Randomness excludes critical features; can repeatedly pick correlated features, which reduces diversity among learners and weakens ensemble effect	Mechanistic correlation improvement in subtype discrimination
Teeth/LIBS-ANN/[147]	ANN requires large data set, 500 spectra are insufficient for analysis; it is an extension of SVM based on sigmoid functions	Tools (LIME and SHAP) can interpret ANN predictions for medical use
Bones/LIBS-SIMCA/[152]	Non-discriminative model which requires pre-processing and PCA before applying SIMCA, limited accuracy of 58% is attained	Kernel PCA or non-linear relationship kernel SIMCA can be used for biomedical data

**Table 3:** Instrumentation and environmental conditions employed for LIBS measurements of different types of cancer. Laser parameters are wavelength, repetition rate, pulse duration, energy per pulse, and fluence. Spectrometer parameters are name/model/available information, spectral resolution, and spectral coverage range. Detector parameters are gate delay and integration time. The environment, type of cancer, and reference are included. Asterisk symbol (*) indicates cancer research was conducted on non-human samples.

Ref.	Laser (Parameters)	Spectrometer	Spectral Resolution	Spectral Range	Gate Delay	Integration Time	Environment	Cancer Type
[150]	Nd:YAG (1064 nm, 1 Hz, 10 ns, 30 mJ)	Avantes AvaSpec 2048	0.20–0.30 nm	190–1100 nm	1.28 µs	1.05 ms	Air	Skin
[145]	Nd:YAG (532 nm, 5 Hz, 5 ns, 7.49 mJ)	Multichannel Instruments	0.1 nm	197–1045 nm	0.2 µs	1.05 ms	Argon	Skin
[48]	Nd:YAG (1064 nm, 5 Hz, 8 ns, 73 mJ)	Avantes AvaSpec ULS2048-4	0.09–0.22 nm	200–850 nm	5 µs	-	Air	Skin, Blood
[118]	Nd:YAG (1064 nm, 1 Hz, 5 ns, 64 mJ)	Avantes AvaSpec 2048-2-USB2	0.2–0.3 nm	190–1100 nm	1.28 µs	2 ms	Air	Skin
[51]	Nd:YAG (532 nm, 20 Hz, 10 ns, 7.49 mJ)	Czerny Turner (SR-500i-B2-R)	-	275–775 nm	0.5 µs	-	Ar	Skin
[117]	Nd:YAG (266 nm, 50 Hz, 8 ns, 8 mJ)	Czerny Turner	-	240–407 nm	0.3 µs	-	Ar	Skin
[52]	Nd:YAG (1064 nm, 4 ns)	Single channel	0.7 nm	270–800 nm	-	1 ms	Air	Skin
[168]	Ti:Sapphire (775 nm, 150 fs, 1.20 mJ & 1.44 mJ)	Echelle	-	-	50 ns	700 µs	Helium	Skin *
[125]	Ytterbium (1030 nm, 550 fs, 250 µJ)	-	0.1 nm	200–900 nm	0.1 µs	-	Ar	Skin *
[82]	Ytterbium (1030 nm, 343 nm, 550 fs, 30–80 µJ, 7.42 J/cm^2^)	Single spectrometer	0.4 nm	240–800 nm	20 ns	-	Air	Skin *
[175]	Nd:YAG (532 nm, 10 Hz, 103 mJ)	Avantes Ava Spec 2048	0.08 nm	190–770 nm	1 µs	2 ms	Air	Breast
[162,163]	Nd:YAG (532 nm, 1 Hz, 98.6 mJ)	Avantes AvaSpec ULS4096CL-Evo	-	200–900 nm	2 µs	-	Air	Breast
[35,47]	Nd:YAG (1064 nm, 1 Hz, 10 ns, 150 mJ)	Avantes Ava Spec 2048	0.4 nm	200–1100 nm	1.28 µs	-	Air	Breast, Colon, Larynx, Tongue
[169]	Nd:YAG (1064 nm, 10 Hz, 6 mJ)	Avantes	-	182–600 nm	2 µs	-	Ar	Breast *
[81]	Ti:Sapph. (785 nm, 1 KHz, 30 fs, 7 µJ)	L.O.T. Oriel Multispec MS125	1 nm		23 ns	-	Air	Breast, Liver
[158]	Nd:YAG (532 nm, 10 Hz, 8 ns, 175 mJ)	Avantes AvaSpec ULS4096CL-EVO	-	200–950 nm	2 µs	-	Air	Lungs, Esophageal
[160]	Nd:YAG (1064 nm, 10 Hz, 10 ns, 65 mJ)	Mechelle Me5000		200–850 nm	1 µs	1 µs	Air	Lungs
[159]	Nd:YAG (1064 nm, 10 Hz, 10 ns, 65 mJ)	Mechelle Me5000	-	200–850 nm	1 µs	1 ms	Air	Lungs
[164]	Nd:YAG (1064 nm, 10 Hz, 40 mJ)	-	-	240–850 nm	6 µs	-	Air	Lungs
[121]	Nd:YAG (1064 nm, 10 Hz, 5 ns, 50 mJ)	Mechelle Me5000	-	200–900 nm	3 µs	-	Air	Lungs
[157]	Nd:YAG (10 Hz, 10 ns, 40 mJ)	-	-	200–900 nm	3 µs	-	Air	Lungs
[166]	Nd:YAG (532 nm, 10 Hz, 8 ns, 30 mJ)	Echelle	-	200–950 nm	1 µs	-	Air	Blood
[119]	Nd:YAG (1064 nm, 5 Hz, 8 ns, 73 mJ)	Avantes AvaSpec ULS2048-4	0.09–0.22 nm	200–850 nm	5 µs	-	Air	Blood
[50]	Nd:YAG (266 nm, 20 Hz, 8 ns, 50 mJ)	SR 500i-A	-	280–900 nm	500 ns	-	Air	Colon
[120]	Nd:YAG (1064 nm, 10 Hz, 10 ns, 20–30 mJ)	Mechelle Me5000	-	200–975 nm	300 ns	-	Air	Colon
[123]	Nd:YAG (1064 nm, 1 Hz, 6 ns, 30 mJ)	Echelle (Kestrel, SE200)	-	200–800 nm	1 µs	-	Air	Stomach
[31]	Nd:YAG (532 nm, 5 Hz, 8 ns, 30 mJ)	Mechelle Me5000	-	200–900 nm	0.9 µs	1 s	Air	Cervical
[161]	Nd:YAG (1064 nm, 10 Hz, 6 ns, 50 mJ)	Echelle (Aryelle 200)	-	193–840 nm	-	-	Air	Cervical
[27]	Nd:YAG (1064 nm, 7 ns, 30 mJ)	Mechelle Me5000		230–900 nm	0.8 µs	-	Air	Ovarian
[45]	Ti:Sapphire (775 nm, 150 fs, 1.6 mJ)	Mechelle Me5000	-	-	50 ns	700 µs	Air	Ovarian
[172]	Ti:Sapphire (775 nm, 150 fs, 1.54 mJ)	Mechelle Me5000	0.013–0.056 nm	220–850 nm	50 ns	700 µs	Helium	Ovarian*
[151,165]	Nd:YAG (1064 nm, 1 Hz, 5 ns, 40 mJ)	Avantes AvaSpec 2048-2-USB2	0.2–0.3 nm	190–1100 nm	1.29 µs	2 ms	Air	Brain
[131]	Nd:YAG (1064 nm, 1 Hz, 10 ns, 50 mJ)	Avantes AvaSpec 2048	0.4 nm	200–1100 nm	1.2 µs	2 ms	Air	Brain
[39]	Nd:YAG (1064 nm, 1 kHz, 7 ns, 270 µJ)	Five channels	-	187–887 nm	0.5 µs	-	Air	Gallbladder
[122]	Nd:YAG (1064 nm, 10 Hz, 8 ns, 40 mJ)	Czerny-Turner	0.3 nm	250–800 nm	2 µs, 10 1 µs	-	Air	Prostrate
[153]	Nd:YAG (1064 nm, 10 Hz, 8 ns, 270 µJ)	-	-	127–868 nm	-	-	Air	Oral
[132]	Nd:YAG (1064 nm, 10 Hz, 6 ns, 8 mJ)	Mechelle (Me5000), Czerny-Turner	0.05 nm, 0.1 nm	250–900 nm	900 ns	-	Air	Oral

**Table 4:** Comparison of laser-induced breakdown spectroscopy (LIBS) with other spectroscopic and optical methods in skin cancer diagnosis based on purpose of study, sample preparation (SP) method, type of analysis (TA), associated biomarkers, and limitations [48,52,117,118,125,145,171,174,182,183,184,185,186,187,188].

Laser-Induced Breakdown Spectroscopy (LIBS)				Other Optical/Spectroscopical Modalities			
Objectives	SP/TA	Biomarkers	Remarks	Objectives	SP/TA	Biomarkers	Remarks
Diagnostic accuracy of LIBS-DNN for skin cancer	No/in vivo	Higher intensities of Ca, Na, and Fe for cancerous tissues	Use of limited dataset, further clinical studies required	Improvement in skin cancer detection by RS-DLM	No/in vivo	Higher line intensities at certain wavenumbers for cancer tissues	Binary classification restricted to exploring staging of disease
Evaluation of LIBS-Raman data fusion method in melanoma diagnosis	FFPE/in vitro	Higher intensities (Ca, Mg), alterations in Raman bands for cancer tissues	Assumption-based study performed only on two subjects	Morpho-chemical characterisation of skin cancer using LC-OCT and CRM	No/ex vivo	Higher intensities of SCC at 821, 1012, 1220, 1446, 1580, 2931 cm^−1^	Fluctuation of intensity for the same tissue, same band shift for different pathologies
* Identification of melanoma lesions from surrounding dermis	Embedded and slicing/in vitro	Higher intensities (Ca, Mg) in affected tissues	Injection of melanoma into mice, cannot apply to human studies	Melanoma cell identification from melanocyte cells by RS	Incubation and centrifugation/in vitro	Higher intensities of Melanoma cells at 645, 947, 1030, 1453, 1582 cm^−1^	Sample preparation involved; limited number of samples being used
LIBS imaging of cutaneous tumours correlated with LA-ICP-MS imaging	FFPE/invitro	Higher Ca and Mg content in tumour region	Poor spatial resolution due to ablation; low sensitivity due to matrix effect and self-absorption	LA-ICP-MS elemental imaging of cutaneous tissue as a complementary method	Tissues on glass slide/in vitro	High intensities of Ca, Mg, P, and Zn in tumour tissues	Spatial and in-depth resolution limited, ablation heterogeneous and destructive, matrix effects
Spectral analysis using ML algorithms for classification of melanoma stages	FFPE/in vitro	P, Ca, Mg, K	Semi-destructive nature of LIBS restricts in vivo analysis	DMF images of skin lesions processed by AI models to achieve diagnostic accuracy	No/in vivo	Cancer-induced alteration in signals of molecules (melanin and keratin)	Accuracy of diagnostic model not high, time-consuming procedure
Classification of melanoma, lymphoma, and healthy subjects based on LIBS-ML algorithms	Serum drops on dry filter paper/in vitro	Ca, Na, K, H, O, N	Reproducibility issues due to shot-to-shot fluctuations, use of filter paper causes uncertainties	Multiphoton microscopy with DL model for diagnostic information on non-melanoma cancer	FFPE/in vitro	MPM images exhibit distinct features for both healthy and abnormal surfaces	Results not very reliable, cross-validation is required
Diagnosis of subtype of melanoma malignancies using LIBS elemental imaging	FFPE/in vitro	Ca, Mg	Small sample size (17) used to classify six subtypes of melanoma	CLSM image classification for diagnostic prediction of skin cancer	Staining procedure/in vitro	Alteration in image processing for two groups of normal and SCC cells	Training of technicians required due to complex and extensive diagnostic procedure
* Examine the melanoma malignancy using fs-LIBS elemental imaging	Frozen sectioning/in vitro	Ca, Mg	Variation of line intensities (plasma fluctuation); low spatial resolution	Processing of FTIR hyperspectral images to classify skin tumour cells	Cells grown on crystal surface/Ex vivo	Malignant cell lines grow disordered, skin cells are flattened	Misclassification by environmental water vapour interference and artefacts

Symbol (*) indicates cancer study performed on non-human specimens. FFPE: formalin-fixed, paraffin-embedded; RS-DLM: Raman spectroscopy and deep learning models; LC-OCT: line-field confocal optical coherence tomography; RS: Raman spectroscopy; CRM: confocal Raman microscopy; LA-ICP-MS: laser-ablation inductively coupled plasma mass spectroscopy; DMF: dermatofluorscopy; CLSM: confocal laser scanning microscopy; FTIR: Fourier transform infrared spectroscopy.

**Table 5:** Summary of recent research works on breast cancer diagnosis by LIBS and other techniques considering study objectives, sample preparation (SP) method, type of analysis (TA), outcomes, and challenges.

Methods	Objectives	SP/TA	Findings	Challenges	Ref.
Laser-induced breakdown spectroscopy (LIBS)	Identify cancerous tissues (breast, colon, larynx, and tongue) from normal ones.	Tissues kept in formalin, cut in slices of ~5 × 5 × 2 mm^3^ /in vitro	Higher plasma temperature for cancerous tissues due to presence of trace elements	Errors in measuring plasma parameters cause uncertainty in element quantification	[35,47]
Laser ablation inductively coupled plasma time of flight mass spectroscopy (LA-ICP-TOF–MS)	Comparative analysis of LA-ICP-TOF-MS and histological staining for breast cancer investigation	Paraffin-embedded breast tissues/in vitro	Both methods revealed elevated levels of Cu, Zn, Sr, and Ba in abnormal tissues	Calibration range 0–33 µg g^−1^, limit of quantification for various metals 11–83 ng g^−1^.	[193]
LIBS and Fourier transform infrared spectroscopy (LIBS-FTIR)	LIBS and FTIR data fusion correction of batch effects in serum spectra for accurate classification of breast cancer using GRAN framework	Centrifugation and storage of the serum sample/in vitro	Superior feature extraction from combined elemental and molecular spectra by CNN model improved diagnosis by mitigating batch effects	Different sample compatibility, computational complexity, concern about reproducibility of results, lack of interpretation of an observed effect	[163]
Particle-induced X-ray emission (PIXE)	Measuring alteration of trace elements in breast cancer patients undergoing chemotherapy and restoration to normal levels after chemotherapy	Centrifugation and freezing of serum samples/in vitro	Elevation of Ca, Cr, Fe, and Cu and alleviation of Ti, Zn, and Se in breast cancer tissues	Requires solid sample, light elements not detected, high uncertainty in quantification heterogeneous samples	[194]
X-ray fluorescence (XRF), X-ray absorption spectroscopy, wide-angle X-ray scattering	Determining the degree of microcalcification (MC) and trace elements association with breast cancer malignancies	FFPE/in vitro	Irregular hydroxyapatite crystal arrangement and distribution of trace elements (Na, S, Cl, Sr and Y) of cancer-affected tissues	Difficult to detect light elements, overlapping of spectral peaks, requirement of matrix match standards	[195]
Inductively coupled plasma mass spectroscopy (ICP-MS), Inductively coupled plasma optical emission spectroscopy (ICP-OES)	Quantification of variation in elemental composition in blood samples of patients	Digestion in microwave oven/in vitro	Reduction in Se and Cr and elevation in Na content in blood of breast cancer patients	Digestion of sample in acid, unable to detect light elements and to quantify halogens, spectral drift; spectral interferences	[196]

**Table 6:** Overview of LIBS studies on various types of cancer, including sample size and spectral analysis information (number of measured spectra, data pre-processing, measurement of plasma parameters, and adoption of AI models).

Cancer Type and Ref.	Sample Preparation	Sample Size	Number of Spectra	Data Preprocessing	Plasma Parameters	Dimensionality Reduction	Model	Model Accuracy
Blood and skin [48]	✓	Moderate	Moderate	✓	✕	✓	KNN	High
Skin [118]	✓	-	Small	✓	✕	✕	ANN/PLS-DA	High
Skin * [150]	✓	Moderate	Small	✓	✕	✓	BP_AdaBoost	High
Skin [52]	✓	Large	Large	✓	✕	✓	DNN	-
Skin * [168]	✓	-	-	✓	✓	✓	Gradient boosting	High
Breast [163]	✓	Large	Small	✓	✕	✕	CNN	Low
Breast [175]	✓	Large	Moderate	✓	✕	✓	SVM	Low
Breast [162]	✓	Large	Moderate	✓	✕	✓	PSCNN	High
Breast/liver [81]	✓	Small	Large	✓	✕	✓	RF/ANN	High
Breast [35]	✓	Large	Large	✓	✓	✕	✕	✕
Breast/colon/larynx/tongue [47]	✓	Large	Large	✓	✓	✕	✕	✕
Lungs/liver/oesophageal [158]	✓	Large	Moderate	✓	✕	✓	SVM	Moderate
Lungs [121]	✓	Large	Small	✓	✕	✕	XGBoost	Low
Lungs [159]	✓	Large	Small	✓	✕	✓	PCA-Boosting tree	High
Lungs [164]	✓	Large	Small	✓	✕	✓	RF-1D ResNet	High
Lungs [160]	✓	Moderate	Small	✓	✕	✓	KNN	High
Lungs [157]	✓	Moderate	Small	✓	✕	✓	KPCA-SVM	High
Blood [166]	✓	Moderate	Small	-	✕	-	RSM-LDA	High
Blood [119]	✓	Moderate	Moderate	✓	✕	✓	LDA/KNN	High
Brain [131]	✓	-	-	-	✓	✕	✕	✕
Brain [165]	✓	Small	Small	✓	✕	✓	SVM	High
Brain [151]	✓	Small	Small	✓	✕	✓	SNN	Moderate
Colon [120]	✓	Small	Small	✓	✓	✕	✕	✕
Colon [50]	✓	Small	Small	✓	✓	✕	✕	✕
Stomach [123]	✓	Small	-	✓	✕	✕	✕	✕
Stomach [156]	✓	Small	Small	✓	✕	✓	SVM/KNN/PLS-DA	High
* Ovarian [172]	✓	Large	Large	✓	✕	✕	RF	Low
Ovarian [27]	✓	Large	Moderate	✓	✕	✓	BPNN	-
Cervical [31]	✓	Small	Small	✓	✕	✓	PCA-SVM	High
Oral [153]	✓	Small	Moderate	✓	✕	✕	LR	-

Sample size: small 1–20, medium 21–50, large > 50. Number of LIBS spectra: small < 3000, medium 3001–15,000, large > 15,000. Model accuracy: low < 80%, moderate 80–90%, high > 90%. Data pre-processing includes baseline correction, normalisation, and standardisation. Plasma parameters are plasma temperature, electron number density, and plasma frequency. Dimensionality reduction by principal component analysis and feature selection methods. Symbol (*) indicates cancer studies performed on non-human samples.

**Table 7:** Overview of AI models applied on LIBS data from measurements of cancer (diagnosis, screening, and staging). LIBS measurements of different samples and types of cancer. Model classification performance metrics are accuracy, sensitivity, specificity, receiver operating characteristic ROC (area under curve AUC), and cross-validation. Symbol (*) indicates cancer studies performed on non-human samples.

AI Model/Sample	Accuracy (%)	Sensitivity (%)	Specificity (%)	ROC Curve (AUC)	Cross-Validation	Cancer Type, Ref.
PCA-KNN/Serum	96	97	95.6	0.99	10-folds	Blood [48]
PCA-KNN/Serum	96	89.2	99.4	0.986	10-folds	Skin [48]
DNN/skin tissues	-	94.6	88.9		10-folds	Skin [52]
PCA-LDA/pellets for melanoma	-	99.4	100	-	10-folds	Skin * [145]
PCA-LDA/excised tissues of melanoma	-	96.7	99.7	-	10-folds	Skin * [145]
BP_AdaBoost/serum for early screening	86.1	-	-	-	10-folds	Skin * [150]
BP_AdaBoost/serum for staging	96.1	-	-	-	10-folds	Skin * [150]
ANN/melanoma FFPE	100	100	100	1	-	Skin [118]
PLS-DA/melanoma FFPE	100	100	100	1	-	Skin [118]
Gradient boosting/Serum on Cu substrate	96.3	-	-	-	5-folds	Skin * [168]
CNN/serum (batch 2)	59.9	0.48	0.71	0.64	-	Breast [163]
GRAN/serum (batch 2)	89.7	0.99	0.80	0.950	-	Breast [163]
Narrow NN/whole blood	91.7	97.2	87.5	0.93	10-fold	Breast [175]
Decision fine Tree/serum	89.7	95.2	83.3	0.87	10-fold	Breast [175]
PSCNN/blood plasma	90	86	94	0.95	5-folds	Breast [162]
RF, ANN, KNN/breast and liver tissue on quartz glass substrate	>94	-	-	-	10-folds	Breast Liver [81]
BVF/serum samples on silicon substrate	92.53	92.92	-	-	-	Lungs Liver Esophageal [158]
CNN/lung tissues	99.17	99.17	99.88	1	-	Lungs [121]
RF boosting tree/lung tissues	98.9	99.3	98.6	0.982	10-folds	Lungs [159]
RF-1D ResNet/lung tissues	91.1	91.3	91.3	0.99	-	Lungs [164]
Bagged tree/tumour and normal tissues	98.9	98.6	99.3	0.982	10-folds	Lungs [160]
KPCA-SVM/tumour and normal tissues	99.03	99.72	98.89	0.970	-	Lungs [157]
RSM-LDA/serum	91	-	-	-	-	Blood [166]
PCA-LDA/blood	99.78	99.6	99.8	1	10-folds	Blood [119]
PCA-KNN/blood	99.72	99.7	99.7	1	10-folds	Blood [119]
FS-SVM/glioma and infiltrative tissue samples	95	-	-	-	-	Brain [165]
SNN/tumour tissues	88.62	-	-	-	-	Brain [151]
BPNN/blood	-	71.4	86.5		-	Ovarian [27]
CNN/cervical cancer cells on silicon wafer	97.92	-	-	-	-	Cervical [161]
PCA-SVM/cervical tissues embedded in paraffin wax	94.4	-	-	-	-	Cervical [31]
PCA-NN/prostate tissue microarrays	97	-	-	-	5-folds	Prostrate [122]
PCA/blood and biological fluids on superhydrophobic (PDMS) substrate	-	88	96	-	-	Oral [132]

## Data Availability

Data will be available on request.

## Abbreviations

The following abbreviations are used in this manuscript:

AAS	Atomic absorption spectroscopy
AdaBoost	Adaptive Boosting
AFM	Atomic force microscopy
AI	Artificial intelligence
ANN	Artificial neural network
AUC	Area under curve
BPNN	Back propagation neural network
BP_AdaBoost	Backpropagation neural network with adaptive boosting
BVF	Bagging voting fusion
BT	Boosting tree
BCC	Basal cell carcinoma
CART	Classification and regression tree
CC-LIBS	Calibration curve LIBS
CF-LIBS	Calibration-free LIBS
CLSM	Confocal laser scanning microscopy
CNN	Convolutional neural network
CRM	Confocal Raman microspectroscopy
CT	Computed tomography
DCS	Dual comb spectroscopy
DFA	Discriminant function analysis
DL	Deep learning
DLM	Deep learning model
DMF	Dermatofluoroscopy
DNN	Deep neural network
DP-LIBS	Dual/double pulse LIBS
EDS/EDX	Energy dispersive X-ray spectroscopy
ED-XRF	Energy dispersive X-ray fluorescence
EF-LIBS	Electric field-assisted LIBS
EPA	Environmental Protection Agency
Er: YAG	Erbium-doped yttrium aluminium garnet
ELISA	Enzyme-linked immunosorbent assay
FDA	Food and Drug Administration
FFF	Front face fluorescence
FFPE	Formalin-fixed, paraffin-embedded
Fs	Femtosecond pulses
Fs-LIBS	Femtosecond LIBS
FS-SVM	Feature selection followed by support vector machine
Fs-DP-LIBS	Femtosecond double-pulse LIBS
FTIR	Fourier transform infrared
GIST	Gastrointestinal stromal tumour
GRAN	Gradient reversal adversarial network
HA	Hydroxyapatite
HAZ	Heat-affected zone
H&E	Hematoxylin and eosin
HSA	Human serum albumin
IB	Inverse bremsstrahlung
ICP-MS	Inductively coupled plasma mass spectroscopy
IL	Intuition learning
IR	Infrared
KNN	Kernel nearest neighbour
KPCA	Kernel principal component analysis
KPCA-SVM	Kernel principal component analysis followed by support vector machine
KW	Kruskal–Wallis
LA-ICP-MS	Laser ablation inductively coupled plasma mass spectroscopy
LA-ICP-TOF–MS	Laser ablation mass spectroscopy, inductively coupled time of flight mass spectroscopy
LC-OCT	Line-field confocal optical coherence tomography
LDA	Linear discriminant analysis
LIBS	Laser-induced breakdown spectroscopy
LIBS-FTIR	Laser-induced breakdown spectroscopy and Fourier transform infrared
LIBS-ML	LIBS-integrated machine learning
LIBS-RS	LIBS-integrated Raman spectroscopy
LIF	Laser-induced fluorescence
LIP	Laser-induced plasma
LOD	Limit of detection
LR	Logistic regression
LTE	Local thermodynamic equilibrium
MCC	Merkel cell carcinoma
Med	Medical
ML	Machine learning
MMG	Mammography
MLA	Machine learning algorithm
MPM	Malignant pleural mesothelioma
MRI	Magnetic reasoning imaging
Nd:YAG	Neodymium-doped yttrium aluminium garnet laser
NE-LIBS	Nanoparticles enhanced LIBS
Ns-LIBS	Nanosecond LIBS
NN	Neural network
OCT	Optical coherence tomography
OLCF	One-line calibration free
PA	Photoacoustic
PAS	Photoacoustic spectroscopy
PCA	Principal component analysis
PCA-KNN	Principal component analysis followed by kernel nearest neighbour
PCA-LDA	Principal component analysis followed by linear discriminant analysis
PDMS	Polydimethylsiloxane
PET	Positron emission tomography
PIXE	Particle-induced X-ray emission technique
PLS-DA	Partial least squares discriminant analysis
PSCNN	Parallel spectral convolutional neural network
Ps-LIBS	Picosecond LIBS
QDA	Quadratic discriminant analysis
ResNet	Residual network
RF	Random forest
RF-1D-ResNet	Radio frequency one-dimensional residual network
RL	Reinforcement learning
ROC	Receiver operating characteristics
RS	Raman spectroscopy
RS-DLM	Raman spectroscopy-deep learning model
RSM	Refined spatial module
RSM-LDA	Refined spatial module-linear discriminant analysis
SA	Self-absorption
SA-LIBS	Spark assisted LIBS
SBR	Signal-to-background ratio
SC	Self-calibrated
SCC	Squamous cell carcinoma
SD-LIBS	Spark discharge LIBS
SEN-LIBS	Surface-enhanced LIBS
SEM	Scanning electron microscopy
SIMCA	Soft independent modelling by class analogy
SKB	Selectkbest
SML	Supervised machine learning
SNN	Spiking neural network
SNR	Signal-to-noise ratio
SOM	Self-organising map
SP	Sample preparation
SP-LIBS	Single pulse LIBS
SVM	Support vector machine
TA	Type of analysis
ULISA	Upconversion-linked immunosorbent assay
Us-LIBS	Ultrashort LIBS
UV-Fs-LIBS	Ultraviolet femtosecond LIBS
UV-NIR	Ultraviolet to near infrared
VUV	Vacuum ultraviolet
WHO	World Health Organization
XGBoost	Extreme gradient boosting
XRF	X-ray fluorescence
